# Recurrent ZC3H18 mutations stabilize oncogenic endogenous retroviral RNA

**DOI:** 10.1016/j.celrep.2026.117526

**Published:** 2026-06-24

**Authors:** Tanzina Tanu, Anna M. Cox, Jennifer A. Karlow, Priyanka Sharma, Kaiwen Sun, Xueyang He, Swathy Babu, Madeline K. Roelofs, Ethan Jeon, Jiajia Chen, Constance Wu, Jared Brown, Nelson B. Barrientos, Andrew S. McCallion, Kevin M. Brown, Stephen J. Chanock, David Liu, Tongwu Zhang, Kathleen H. Burns, Paul L. Boutz, Megan L. Insco

**Affiliations:** 1 Department of Medical Oncology, Dana-Farber Cancer Institute, Boston, MA 02115, USA; 2 Department of Pathology, Dana-Farber Cancer Institute, Boston, MA 02215, USA; 3 University of Rochester School of Medicine and Dentistry, Rochester, NY 14642, USA; 4 Center for RNA Biology, University of Rochester, Rochester, NY 14642, USA; 5 Wilmot Cancer Institute, Rochester, NY 14642, USA; 6 Stem Cell Program and Division of Hematology/Oncology, Boston Children’s Hospital, Boston, MA 02115, USA; 7 Department of Data Science, Dana-Farber Cancer Institute, Harvard Medical School, Boston, MA 02115, USA; 8 McKusick-Nathans Department of Genetic Medicine, Johns Hopkins University School of Medicine, Baltimore, MD 21205-1832, USA; 9 Division of Cancer Epidemiology and Genetics, National Cancer Institute, Bethesda, MD 20850, USA; 10 These authors contributed equally; 11 Lead contact

## Abstract

Endogenous retroviral (ERV) RNA is highly expressed in cancer, suggesting a selective advantage. We identify recurrent truncating mutations in ZC3H18 (Z18), a nuclear RNA surveillance factor lost in 30% of all cancers. We show that Z18 truncating mutations are oncogenic and that Z18 plays an evolutionarily conserved role in the nuclear surveillance of oncogenic ERV RNA. In zebrafish, Z18 truncation accelerates melanoma onset and selectively increases ERV RNA. Human cancer cell lines and patient tumors with Z18 mutations also upregulate ERV RNA. In engineered human melanoma cells, Z18 truncation enhances ERV RNA accumulation more than heterozygous Z18 loss, consistent with dominant-negative activity. Truncated Z18 directly stabilizes and relocalizes ERV RNA to the cytoplasm. ERV RNA expression accelerates melanoma and is required for Z18 truncation-mediated melanoma onset in zebrafish. Growth of human melanoma cells similarly depends on Z18-regulated ERV RNA. Together, these findings support Z18-regulated ERV RNA as a driver of oncogenesis.

## In brief

Truncating mutations in the nuclear RNA surveillance factor ZC3H18 (Z18) recur across cancers and drive melanoma by stabilizing endogenous retroviral (ERV) RNA. Using zebrafish models, cancer cell lines, and patient tumors, Tanu et al. demonstrate that Z18 truncation or loss stabilizes ERV RNA, which is necessary and sufficient for oncogenesis.

## Graphical Abstract

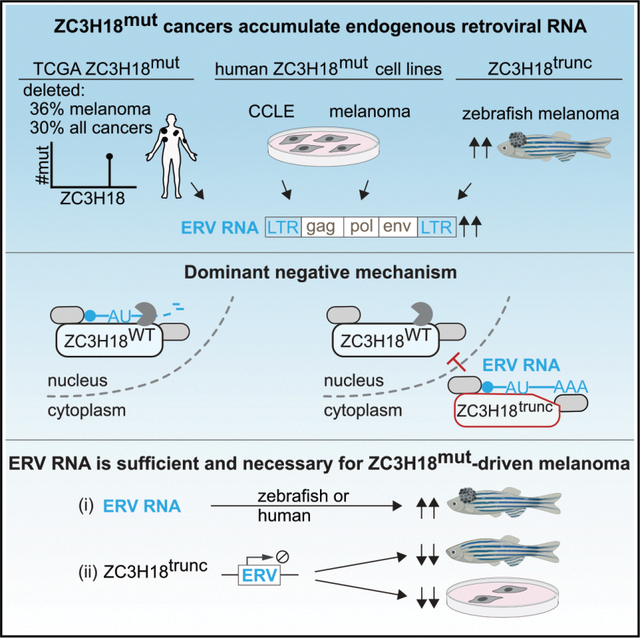

## INTRODUCTION

As healthy cells require appropriate levels of high-quality RNA, dysregulation of RNA homeostasis contributes to diverse human diseases, including cancer. RNA homeostasis requires balanced production and degradation. While the role of RNA production through transcription factors, chromatin regulation, and enhancers in cancer is well studied,^1^ how perturbations in RNA degradation pathways contribute to cancer is less clear. Mechanisms that detect and degrade aberrant or unstable nuclear RNAs have only recently been described,^2–5^ and we previously discovered that the deficient nuclear RNA surveillance of aberrant prematurely terminated RNAs (ptRNAs) is oncogenic.^6^ Nuclear RNA surveillance components are mutated in up to 21% of melanomas, and recurrent mutations occur across cancers in two components, including ZC3H18^6^ (Z18). This pattern of mutations suggests that deficient nuclear RNA surveillance is a widespread contributor to human tumorigenesis.

Different protein complexes target unstable or aberrant nuclear RNA for degradation. The polyA exosome targeting (PAXT) complex targets aberrant polyadenylated transcripts,^4,7^ including oncogenic ptRNAs.^6^ The nuclear exosome targeting (NEXT) complex^3^ identifies a wide range of noncoding RNAs for degradation, including promoter upstream transcripts (PROMPTs), enhancer RNAs (eRNAs),^8^ long interspersed element-1 (LINE-1) retrotransposons,^9^ and long terminal repeat (LTR) retrotransposons, including endogenous retroviral (ERV) RNAs.^10^ Retroviral-derived ERVs and LTRs comprise approximately 8% of the human genome^11^ and have accumulated mutations that render them noninfectious and immobile.^12^ ERV sequences can retain enhancer and polymerase II (Pol II) promoter activity, as well as splice sites and polyadenylation signals,^13,14^ enabling the generation of noncoding RNAs or novel open reading frames either independently or when incorporated into host transcripts.^15^ Transposable elements are upregulated in multiple cancer types, with the ERV class of retroelements being most affected.^16,17^ ERV upregulation in cancer correlates with increased immune infiltration and immunotherapy efficacy in multiple cancers.^17–19^ However, the causes of ERV upregulation in cancer are unknown, and it remains unclear whether elevated levels of ERV transcripts or proteins are pathogenic or merely an epiphenomenon of broader dysregulation in malignant tissue.

In this study, we show that recurrent truncating mutations in Z18 produce a truncated protein that retains its ability to bind RNA and lacks the domain required to recruit RNA degradation machinery. This truncated isoform accelerated melanoma onset in a zebrafish model and specifically promoted the accumulation of ERV RNAs, without affecting the levels of other aberrant or unstable nuclear RNAs. We find that in humans, the role of Z18 in ERV degradation is conserved, that Z18 truncating mutations function in a dominant-negative manner, and that the truncated isoform binds and stabilizes ERV RNA in the cytoplasm. Importantly, expression of zebrafish or human ERV RNA was sufficient to accelerate melanoma in zebrafish, and ERV RNA was required for both mutant Z18-driven melanoma in zebrafish and the growth of human melanoma cells. By studying Z18 mutations in melanoma, we reveal a functional role for ERV RNA in oncogenesis. This study supports that accumulated aberrant RNA contributes directly to cancer phenotypes.

## RESULTS

### Recurrent Z18 truncating mutations are oncogenic

We previously showed that cyclin-dependent kinase 13 (CDK13) activates the PAXT complex, which targets aberrant ptRNAs for degradation. When CDK13 is mutated, ptRNAs are stabilized, leading to more aggressive melanoma.^6^ We decided to investigate Z18, a PAXT component previously identified as a low-frequency driver in melanoma (OncodriveFM).^6^ In The Cancer Genome Atlas (TCGA) melanoma^20^ patient samples, PolyPhen-2^21^ predicted enrichment of deleterious Z18 mutations (*p* = 0.0047). In a larger melanoma cohort (*n* = 1347), 35% of patients with copy-number data had bi-allelic or monoallelic loss (291/823). Z18 deletions were also enriched in the all-cancer dataset (Q = 1.75E^−54^, 30.0% frequency), which includes melanoma (Q = .0001, 36.6% frequency).^22^ Of the 64 melanoma patients with Z18 mutations, 50 (78%) had deleterious mutations (7 truncating and 43 missense) (PolyPhen-2) (Data S1, “Z18 Mut & Melanoma Patient Refs”). Other TCGA cancers also showed significant enrichment of deleterious mutations by PolyPhen-2, including uterine corpus endometrial carcinoma (UCEC) (*p* = 0.0041), cervical squamous cell carcinoma (*p* = 0.028), colon adenocarcinoma (COAD) (*p* = 0.013), and lung adenocarcinoma (*p* = 0.016) (Data S1, “PolyPhen-2 Results”). These data suggest selection of deleterious Z18 mutations and copy-number loss in melanoma and other cancers.

**Figure 1.**
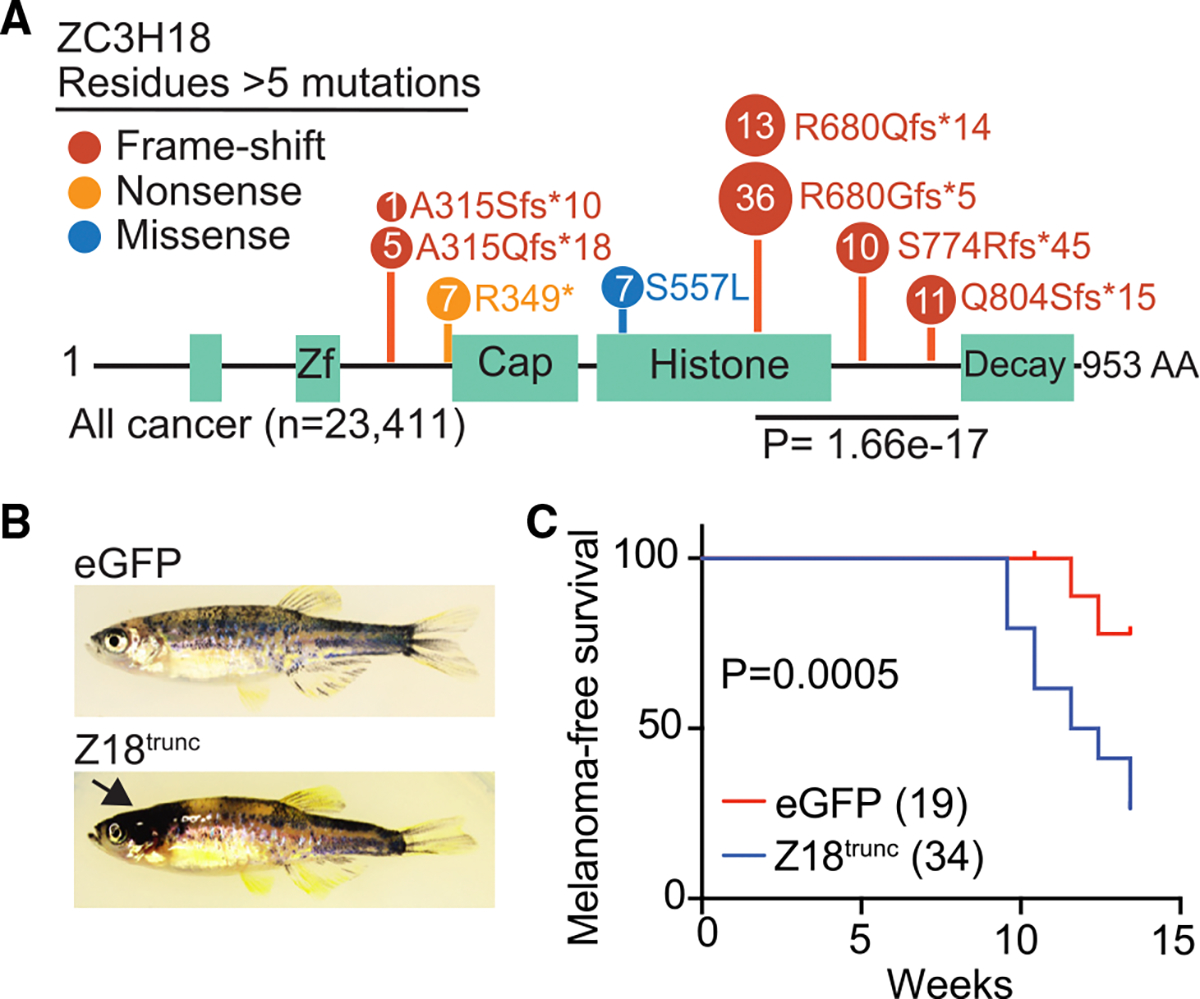
Recurrent ZC3H18 truncating mutations are oncogenic (A) Lollipop plot of Z18 mutations across pan-cancer patients. *p* = 1.66e–17 (Fisher’s exact test; truncating mutations in residues 679–901 versus remainder). *n* = patients. (B and C) Triple-mutant zebrafish with melanocyte-specific eGFP or human Z18^trunc^ (R680Gfs*5). (B) Representative images at 9 weeks. Arrow = early melanoma. (C) Melanoma-free survival. Log rank. *n* = zebrafish. See also Figure S1.

We next analyzed Z18 mutations in a larger pan-cancer cohort (Data S1, “Pan-cancer Patient Sample Refs”). We identified 506 deleterious Z18 mutations from 23,411 patients and found that truncating mutations (Z18^trunc^) were statistically enriched in the region preceding the RNA surveillance complex-binding domain (amino acid 679–901) compared to the rest of the protein (odds ratio = 5.89, *p* = 1.66e–17, Fisher’s exact test) (Figure 1A; Data S1, “Z18^trunc^ Fisher’s exact test” and “Pan-cancer Z18 Mutations”). Truncating mutations clustered at R680 (49 patients) and just downstream (37 patients), suggesting that loss of the distal C-terminal domain confers a selective advantage. Z18^trunc^ mutations were most frequently found in endometrial carcinoma (20/67), stomach adenocarcinoma (18/29), and colorectal adenocarcinoma (14/35). Z18 mutations were enriched in microsatellite instability (MSI)-high tumors, although most occurred in microsatellite-stable cancers (Table S1). Because loss-of-function mutations are expected to be uniformly distributed across a gene, the enrichment of Z18 truncations suggests an alternative function, such as dominant-negative activity. The Z18 C-terminal domain is essential for recruiting RNA surveillance machinery.^23^ Thus, we hypothesized that these mutations create an oncogenic isoform that cannot degrade target RNAs, thereby promoting cancer.

As human and zebrafish Z18 are highly similar (67.62% nucleotide sequence and 62.9% protein sequence using the tool BLAST^24^), we tested the function of the most frequent patient truncating mutation, Z18 R680Gfs*5 (hereafter Z18^trunc^), in zebrafish melanoma (Figure S1A), using the MAZERATI rapid genetic modeling system in *BRAF*^*V600E*^*; p53*−/−*; mitfa*−/− zebrafish (triple-mutant),^25–27^ which allows melanocyte-specific genetics in first-generation animals. Z18^trunc^ expression produced more black patches (early melanoma) at 9 weeks (Figures 1B and S1B) and expedited melanoma onset compared to control eGFP expression (Figure 1C, replicate in S1C and S1D). Human Z18^trunc^ expression accelerated melanoma onset in zebrafish.

### Z18^trunc^ promotes ERV RNA accumulation

**Figure 2.**
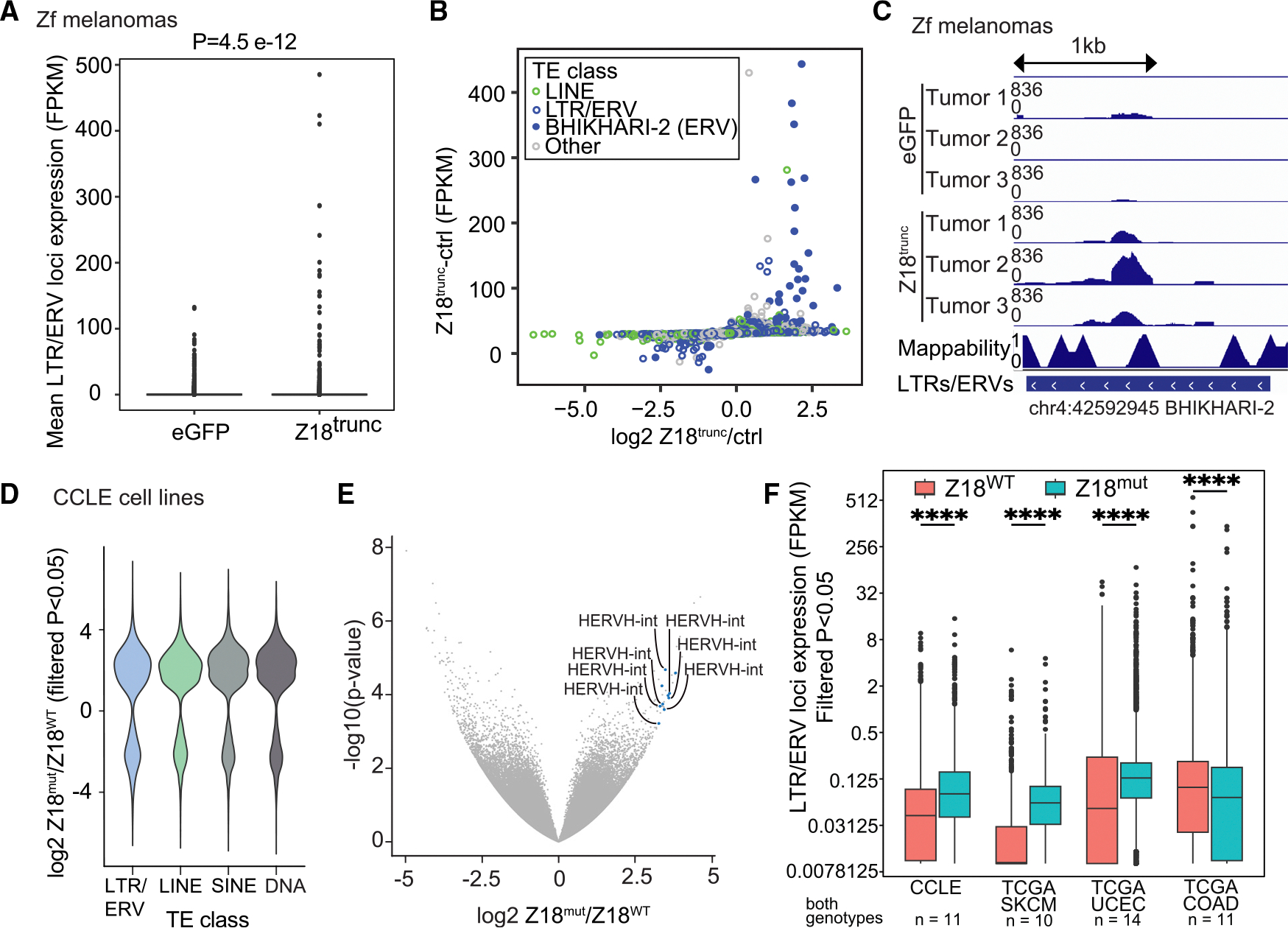
ZC3H18^trunc^ promotes ERV RNA accumulation (A) Mean LTR loci expression from pA-selected RNA-seq of zebrafish melanomas expressing eGFP (*n* = 3) or Z18^trunc^ (*n* = 3). Wilcoxon test. (B) Difference in mean TE locus expression versus log2 fold change for Z18^trunc^ (*n* = 3) relative to eGFP (*n* = 3) melanomas. Circles = individual TE loci. (C) IGV tracks for a representative ERV in eGFP- and Z18^trunc^-expressing zebrafish melanomas. (D) Log2 fold change for significantly altered TE loci (*p* < 0.05, Wilcoxon test) in Z18^mut^ (*n* = 11) versus Z18^WT^ (*n* = 11) CCLE lines. (E) Volcano plot of DESeq2-normalized TE loci (*n* = 271,879 loci) from Z18^mut^ (*n* = 11) versus Z18^WT^ (*n* = 11) CCLE lines. Blue dots = significantly upregulated LTRs/ERVs. HERVH-int elements are labeled. (F) Mean expression of significantly changed LTR/ERV loci (*p* < 0.01, Wilcoxon test) in Z18^WT^ and Z18^mut^: CCLE cell lines (*n* = 11 each, *n* = 5,444 loci), TCGA melanoma patients (*n* = 10 each, *n* = 591 loci), TCGA uterine cancer patients (*n* = 14 each, *n* = 7,252 loci), and TCGA colon cancer patients (*n* = 11 each, *n* = 5,640 loci). ****p < 2.2e–16, Wilcoxon test. In boxplots, the black line marks the median, boxes indicate the interquartile range (IQR), and whiskers extend to 1.5× IQR. See also Figure S2.

We asked whether Z18^trunc^ stabilizes RNA surveillance substrates. Because Z18 binds to the PAXT complex,^4^ which clears polyadenylated ptRNAs,^6^ we quantified ptRNAs from pA-selected bulk RNA sequencing (RNA-seq) from zebrafish tumors with melanocyte-specific expression of eGFP (*n* = 3) or Z18^trunc^ (*n* = 3) (Figure S2A). A curated set of CDK13-dependent ptRNAs was not significantly upregulated (Figure S2B). As Z18 is also reported to associate with the NEXT nuclear RNA surveillance complex,^3^ we assayed NEXT substrates. Transposable element (TE) expression from pA-selected RNA-seq was quantified using a tool called SQuIRE.^28^ Average TE expression was calculated for eGFP (*n* = 3) and Z18^trunc^ melanomas (*n* = 3), revealing a significant increase in LTRs/ERVs in Z18^trunc^-expressing tumors (*p* = 4.5e–12, Wilcoxon test) (Figure 2A). Significant increases were also seen for LINEs and short interspersed elements (SINEs), although the magnitude of change was smaller (Table S2). To identify the most affected TEs, we plotted average expression difference versus fold change (Figure 2B), and the 27 elements with the largest expression difference were inspected for autonomous expression using Integrative Genomics Viewer (IGV).^29^ The most upregulated element was ERV *BHIKHARI-2* (*bhik-2*, also known as *crestin*), which is transiently expressed during neural crest development, required for this process,^30^ and re-expressed during melanoma initiation.^31^ Most of the top upregulated TEs were LTRs/ERVs (79%, 21/27), with *bhik-2* ERVs being most affected (71% of LTRs/ERVs, 14/21, solid blue dots in Figure 2B represent *bhik-2* elements, example in Figures 2B, 2C, and S2C). Most of these TEs were expressed independently of nearby genes (56%, 15/27), including all 14 *bhik-2* elements. To test whether other NEXT substrates were affected by Z18^trunc^ expression, we conducted ribo-minus RNA-seq of Z18^trunc^ (*n* = 3) versus eGFP (*n* = 3) melanomas (Figure S2D). PROMPTs and eRNAs did not accumulate in Z18^trunc^-expressing melanomas (Figure S2E; Table S3). Thus, Z18^trunc^ promotes ERV RNA accumulation without broadly affecting other NEXT targets,^3^ suggesting selective ERV RNA regulation.

Because Z18 is conserved between zebrafish and humans, we asked whether it similarly regulates ERV RNA accumulation in human cell lines. We used SQuIRE to quantify TE expression from publicly available pA-selected RNA-seq from the Cancer Cell Line Encyclopedia (CCLE).^32^ Cell lines with deleterious Z18 mutations (Z18^mut^) (*n* = 11) were compared with matched Z18 wild-type (Z18^WT^) cell lines (cancer-type, sex, driver mutations) (*n* = 11) (Figure S2F; Data S2, “CCLE cell lines”). Average log2 fold change in TE expression was plotted for TEs that were significantly different (*p* < 0.05) between Z18^mut^ and Z18^WT^ cell lines (Figure 2D). All TE classes were upregulated in Z18^mut^ lines relative to controls, with the most regulated elements being LTRs/ERVs (Figure 2D, blue distribution extends to higher values). Using DESeq2^33^ normalization to distribute the data for easier visualization, we found that most of the significantly upregulated TEs were LTRs/ERVs (10/30 upregulated), with seven loci from the HERV-H subfamily (Figure 2E), which manual inspection showed to be derived predominantly from colon cancer lines. We quantified ERV expression and found significant enrichment of LTRs/ERVs in Z18^mut^ relative to Z18^WT^ CCLE cell lines (Figure 2F, left). One significantly upregulated, autonomously expressed ERV at chr16:2659700 was expressed in multiple cancer types, initiated in one element and readthrough several additional annotated TE intervals before terminating (Figure S2G). Thus, Z18-mutant cell lines across cancers upregulate TEs, especially ERVs.

Because no Z18-mutant CCLE melanoma cell lines had available RNA-seq, we analyzed TCGA data to assess Z18’s role in ERV regulation in melanoma. We quantified ERV expression in Z18^mut^ and Z18^WT^ TCGA skin cutaneous melanoma (SKCM) with publicly available pA-selected RNA-seq using SQuIRE. Patient samples with deleterious Z18 mutations (alpha missense >0.564, nonsense, or frameshift) were selected (*n* = 11). Matched Z18^WT^ samples were also selected (stage, sex, age, and driver mutations [BRAF, NRAS, and NF1]) (*n* = 11). This analysis showed significant accumulation of LTRs/ERVs in Z18^mut^ SKCM patients (Figure 2F, left middle; Data S2, “TCGA SKCM patients”). Because Z18 mutations are the most common in UCEC and COAD cancers, we selected TCGA Z18^mut^ cases: UCEC (*n* = 14) and COAD (*n* = 11). We similarly analyzed LTR/ERV expression in the aforementioned datasets and found significant enrichment of LTRs/ERVs in Z18^mut^ relative to Z18^WT^ UCEC patients (Figure 2F, right middle; Data S2, “TCGA UCEC patients”). Global LTR/ERV upregulation was not observed in Z18^mut^ COAD (Figure 2F, right; Data S2, “TCGA COAD patients”), but 50% of the most upregulated TEs in Z18^mut^ COAD were ERVs (15/30), and 53% of these were HERV-H (8/15), the same subfamily found to be upregulated in Z18^mut^ CCLE cell lines, suggesting that Z18 plays a conserved role in suppressing HERV-H elements in colon cancer. Overall, these analyses suggest that Z18-mediated ERV RNA suppression is conserved across species and relevant to multiple cancer types.

### Z18^trunc^ stabilizes ERV RNA in a dominant-negative manner

**Figure 3.**
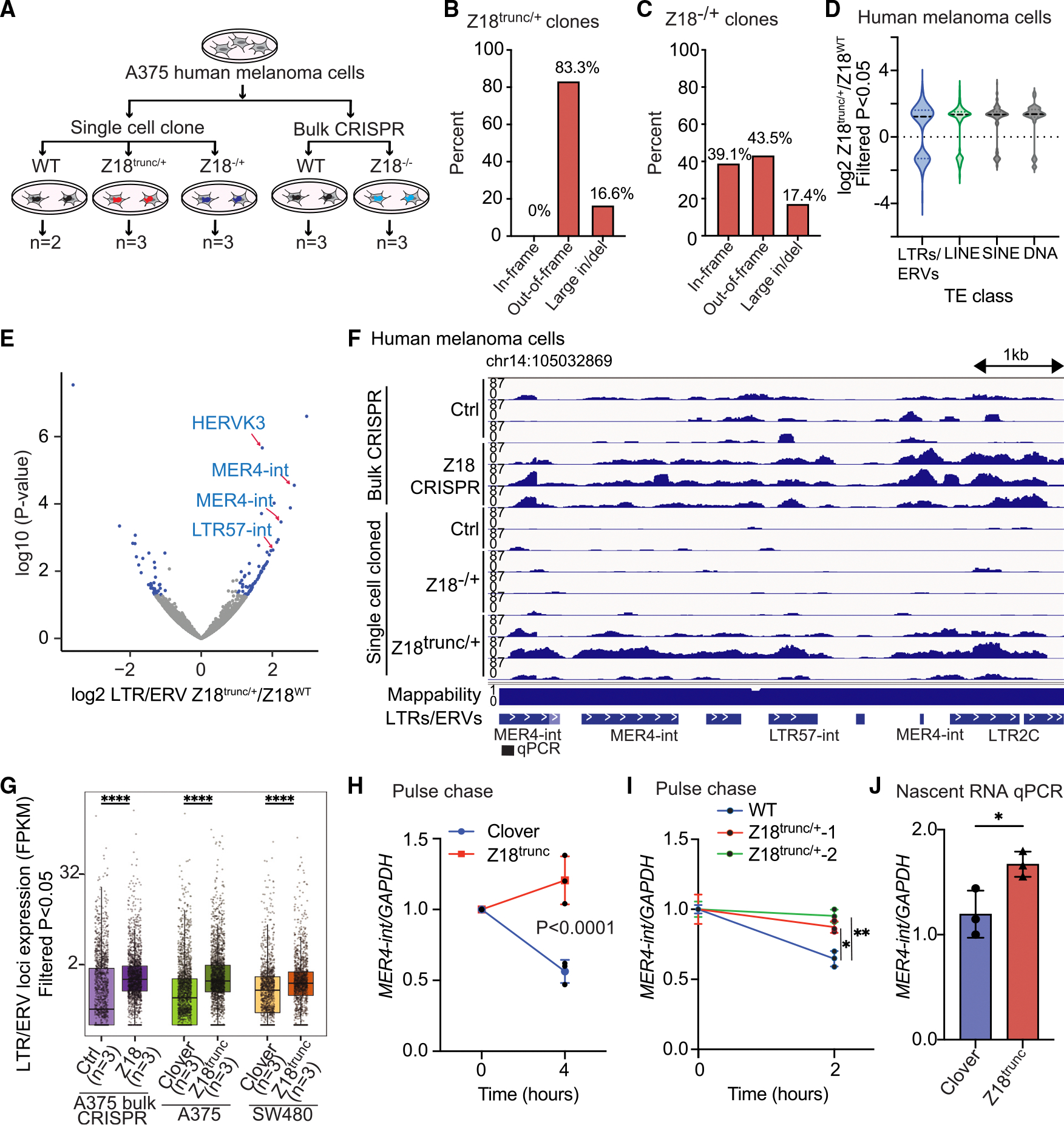
ZC3H18^trunc^ stabilizes ERV RNA in a dominant-negative manner (A) Schematic of the Z18 allelic series generated in A375 human melanoma cells. (B and C) Percent in-frame or out-of-frame Z18 CRISPR mutations for (B) Z18^trunc^ clones (*n* = 24) and (C) Z18 loss-of-function clones (*n* = 23). (D) Log2 Z18^trunc/+^ (*n* = 3) versus Z18^WT^ (*n* = 2) human melanoma cell lines significantly changed TEs (*p* < 0.05) by class. Thick dotted line = median. Light dotted line = quartiles. (E) Volcano plot of LTR/ERV locus expression (*n* = 12,526 loci) from Z18^trunc/+^ (*n* = 3) versus Z18^WT^ (*n* = 2) A375s. Blue dots = significantly changed LTRs/ERVs. (F) IGV tracks of significantly upregulated *MER4-int* LTR/ERV. (G) Mean expression of significantly changed LTR/ERV loci (*p* < 0.01, Wilcoxon test) in bulk Z18 CRISPR (*n* = 3 replicates/genotype, *n* = 1,736 loci), Z18^trunc^-expressing A375 human melanoma cell lines (*n* = 3 lines/genotype, *n* = 2,073 loci), and Z18^trunc^-expressing SW480 human colon cancer cell lines (*n* = 3 lines/genotype, *n* = 1,286 loci). ****p < 2.2e–16, Wilcoxon test. In boxplots, the black line marks the median, boxes indicate IQR, and whiskers extend to 1.5× IQR. (H and I) Decay of *MER4-int* using 4sU pulse-chase qPCR. Primer location in (F). (H) Z18^trunc^- versus Clover-expressing lines (*n* = 3 each). Mean ± SD. Two-way ANOVA. (I) Z18^trunc/+^ (*n* = 2) versus Z18^WT^ (*n* = 1) lines. Mean ± SD. 2 h *p = 0.013 (Z18^trunc/+^ line 1 versus Z18^WT^), **p = 0.0017 (Z18^trunc/+^ line 2 versus Z18^WT^) (two-way ANOVA). (J) qPCR of 4sU-labeled nascent *MER4-int* transcript expression from Clover- and Z18^trunc^-expressing human melanoma cells. Mean ± SD. *p = 0.032 (two-tailed unpaired *t* test). See also Figure S3.

To test whether Z18^trunc^ promotes ERV RNA accumulation, we generated isogenic clonal human melanoma cell lines that were heterozygous for Z18 patient truncating mutations using CRISPR homology-directed repair (HDR) (Z18^trunc/+^, *n* = 3) or Z18^WT^ (*n* = 2) (Figures 3A and S3A–S3E; Table S4). We recovered one HDR-edited Z18^trunc/+^ clone (R680Gfs*5) and two clones harboring other Z18^trunc/+^ patient mutations. To test whether Z18^trunc^ interferes with Z18^WT^, human melanoma cells with Z18 heterozygous loss-of-function mutations (Z18^−/+^) were also generated (*n* = 3) (Figures S3F–S3G). The guide RNA (gRNA) used to make Z18^trunc/+^ clonal cell lines yielded out-of-frame truncating mutations in 83.3% of edited cell clones (20/24; Figure 3B), whereas the two gRNAs used for heterozygous loss-of-function clones resulted in fewer out-of-frame mutations (10/23, 43.5%; Figure 3C; gRNA locations in Figure S3A), suggesting selection for Z18^trunc/+^ mutations. Because no viable homozygous loss-of-function clones were recovered (0/23 clones with mutations), we also performed transient bulk CRISPR (Figures S3H and S3I) to assess the Z18 loss-of-function phenotype.

To determine if Z18^trunc^ regulates ERV RNA in a dominant-negative manner, we used SQuIRE to quantify TEs from pA-selected RNA-seq in the Z18 allelic series. Z18^trunc/+^ upregulated all TE classes relative to Z18^WT^, with LTRs/ERVs most affected (Figure 3D, blue distribution extends to higher values). Bulk Z18 CRISPR-deleted cells also showed upregulation of all TEs, except SINEs (Figure S3J). Given the variability in SINE changes across models, we focused subsequent analyses on ERVs, which showed robust and consistent upregulation across Z18 perturbations. The most upregulated autonomously expressed LTR in the Z18^trunc/+^ cell lines was chr14:105032869 *MER4-int* (hereafter *MER4-int*) (Figures 3E, 3F, and S3K, qPCR primer location; Figure 3F; Table S4). Z18 loss of function via bulk CRISPR deletion also resulted in *MER4-int* upregulation (Figure 3F, top 6 rows; Figure 3G, left). Notably, *MER4-int* accumulated to higher levels in Z18^trunc/+^ cells than in cells with loss of a single Z18 allele (Figure 3F, last six rows), suggesting that Z18^trunc^ interferes with Z18^WT^-mediated ERV RNA clearance and acts in a dominant-negative manner. Z18^trunc/+^ likewise increased another significantly regulated and autonomously expressed LTR/ERV, *HERVK3-int* (chr16:35516910) (hereafter *HERVK3-int*) (Figures S3L and S3M, qPCR primer location S3M; Table S4). These data support dominant-negative ERV RNA accumulation by Z18^trunc/+^.

To assess the impact of Z18^trunc^ mutations on the broader transcriptome, gene set enrichment analysis (GSEA)^34^ was performed using differentially expressed RefSeq transcripts obtained from pA-selected bulk RNA-seq of the Z18^trunc/+^ versus control single-cell clones. Of the Hallmark signatures,^35^ the most significantly upregulated pathway was “TNFA_SIGNALING_VIA_NFKB” (family-wise error rate *p* = 0, normalized enrichment score 2.44) (Figure S3N; Data S3, “MSigDB Hallmark Z18^trunc^ vs. Ctrl”), and the two most upregulated pathways from the Chemical and Genetic Perturbation (CGP) datasets were associated with viral infection, suggesting viral mimicry (“RESPIRATORY_SYNCYTIAL_VIRUS_INFECTION_A594_CELLS_UP” and “HUMAN_PARAINFLUENZA_VIRUS_3_INFECTION_A594_CELLS_UP”) (Figure S3O; Data S3, “CPG Z18^trunc^ vs. Ctrl”). We hypothesize that widespread ERV RNA accumulation induces viral mimicry, as reported for ERV transcriptional derepression.^17,36,37^

Because CRISPR-HDR and single-cell cloning introduce heterogeneity, and because Z18^trunc^ functions in a dominant-negative manner, we generated Z18^trunc^- and Clover-expressing control human melanoma cell lines (Figure S3P). We quantified TE expression from pA-selected RNA-seq and visualized significantly changed (*p* < 0.05) loci. Consistent with our previous findings, multiple TE classes were upregulated, with LTRs/ERVs being the most affected (Figure 3G, middle; Figures S3Q and S3R). A union of significantly changed ERVs/LTRs showed that the magnitude of accumulation versus controls was similar (Figure S3S). CDK13-dependent ptRNAs were unaffected (Figure S3T). To measure the effects of Z18^trunc^ on additional NEXT substrates, we performed ribo-minus RNA-seq on Z18^trunc^- versus Clover-expressing A375s and again found that all TEs were broadly regulated (Figure S3U), with no change in other NEXT targets (Figure S3V). Polyadenylated TEs were more strongly regulated than non-polyadenylated TEs (pA-selected versus rRNA-depleted RNA-seq) (Figure S3W), suggesting that Z18 may preferentially regulate polyadenylated substrates. To test for a functional role in colon cancer, we performed parallel expression of Z18^trunc^ or Clover in SW480 colon cancer cells and found that ERVs/LTRs were significantly upregulated (Figure 3G, right; Figure S3X), although the effect size was smaller, consistent with Z18 playing a more limited role in LTR/ERV regulation in colon cancer.

To determine if the increase in the *MER4-int* transcript was due to defective degradation, we measured its decay kinetics. Using 4sU pulse-chase, we found significant stabilization of *MER4-int* in Z18^trunc^-expressing (*n* = 3) relative to Clover-expressing (*n* = 3) cell lines (Figures 3H and S3Y). *MER4-int* was also stabilized in Z18^trunc/+^ as compared to Z18^WT^ (Figure 3I). To assess *MER4-int* transcription, nascent RNA^38^ qPCR was completed for Clover- and Z18^trunc^-expressing human melanoma cells. Z18^trunc^ cells showed higher *MER4-int* transcription (Figure 3J), consistent with a report that LINE-1 chromatin is more accessible upon depletion of ZCCHC8, a core NEXT component.^9^ These data show that the *MER4-int* transcript is stabilized in Z18^trunc^-expressing and Z18^trunc/+^ cells and suggest that *MER4-int* decay may play a role in its own chromatin silencing. Together, our data are consistent with a model where Z18^trunc^ stabilizes ERV messages in a dominant-negative manner.

### ZC3H18^trunc^ directly promotes oncogenic ERV RNA stabilization

**Figure 4.**
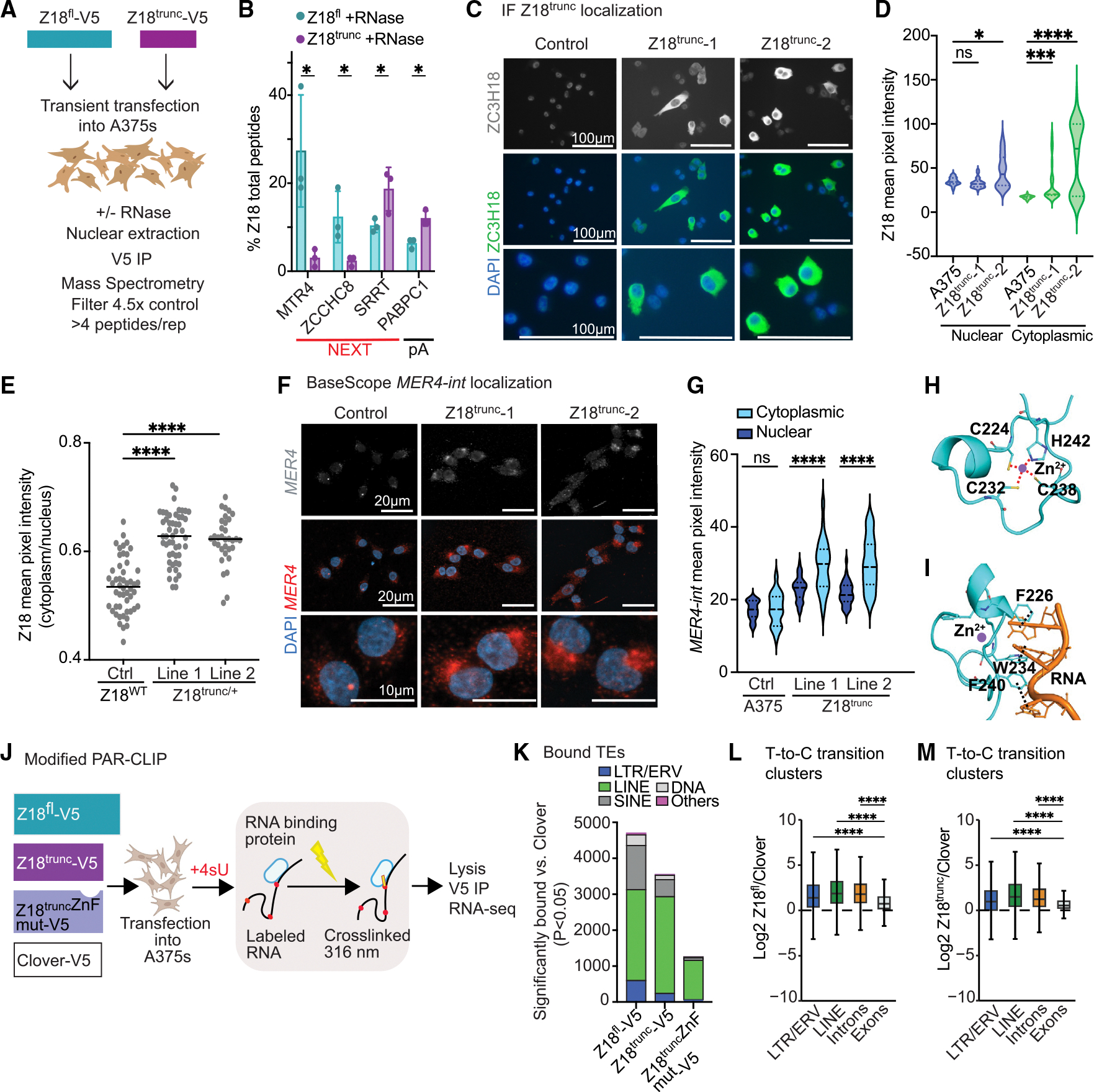
ZC3H18^trunc^ directly promotes oncogenic ERV RNA stabilization (A) Schematic of immunoprecipitation-mass spectrometry (IP-MS). fl, full length. (B) IP-MS total peptides normalized to Z18 for proteins with differential binding between Z18^fl^ versus Z18^trunc^ (+RNase). MTR4 *p = 0.028, ZCCHC8 *p = 0.043, SRRT *p = 0.046, PABPC1 *p = 0.010 (two-sided *t* test). (C and D) Immunofluorescence (IF) for ZC3H18 in control and Z18^trunc^-expressing line1/line2. (C) Representative images. Blue = DAPI (nucleus). Green = ZC3H18. Scale bars, 100 μm. (D) Mean nuclear and cytoplasmic ZC3H18 pixel intensity (*n* = 50 cells/condition). *p < 0.0137, ***p = 0.0002, ****p < 0.0001, ns = non-significant (two-way ANOVA). (E) IF for ZC3H18 in Z18^WT^ and Z18^trunc/+^ line1/line2 mean cytoplasmic to nuclear pixel intensity (*n* = 50, 50, or 33 cells, respectively). ****p < 0.0001 (two-way ANOVA). (F and G) *MER4-int* RNA localization by BaseScope in control (A375) and Z18^trunc^-expressing line1/line2. (F) Representative images. Blue = DAPI (nucleus). Red = *MER4-int*. Scale bars, 20 (row 1 and 2) and 10 μm (row 3). (G) Mean cytoplasmic and nuclear *MER4-int* pixel intensity (*n* = 18, 48, or 35 cells, respectively). ****p < 0.0001; ns = non-significant (one-way ANOVA). (H and I) Modeled Z18 zinc-finger domain (cyan): (H) Zn^2+^ ion (purple) coordinated by three cysteines and one histidine; (I) RNA (orange) predicted pi-pi stacking interactions (dashed lines). (J) Schematic of PAR-CLIP workflow. (K) Significantly bound TEs (*p* < 0.05, DESeq2) from Z18^fl^-V5, Z18^trunc^-V5, and Z18^truncZnFmut^-V5 versus Clover-V5. (L and M) Log2 binding to LTR-, LINE-, intron-, or exon-containing clusters for (L) Z18^fl^ or (M) Z18^trunc^ versus Clover. In boxplots, the black line marks the median, boxes indicate IQR, and whiskers extend to 1.5× IQR. ****p < 0.0001. Medians and two-sided Wilcoxon test *p* values in Table S5. See also Figure S4.

To define the mechanism, we identified proteins that bind full-length (Z18^fl^) and truncated Z18 via immunoprecipitation mass spectrometry (IP-MS). V5-tagged Z18^fl^ and Z18^trunc^ were transiently expressed in A375 human melanoma cells. Nuclear protein was extracted under native conditions, and Z18 was immunoprecipitated (IPed) in triplicate using a V5 antibody. IPs were performed in the presence or absence of RNase A/T1 to define RNA-dependent and -independent protein interactions (Figure 4A). Data were filtered for proteins that were >4.5× enriched over a published control IP^6^ and >4 total peptides per replicate in the most permissive condition (Data S4). Z18^fl^ abundantly bound NEXT components, ZCCHC8 and MTR4 (Figure 4B, left), consistent with our data showing that Z18 regulates ERV RNA turnover, which requires NEXT.^10^

We then compared Z18^fl^- and Z18^trunc^-interacting proteins (Figure S4A). As expected, the loss of the C-terminal RNA surveillance recruitment domain caused a significant loss of MTR4 and ZCCHC8 binding to Z18^trunc^ compared with Z18^fl^ (Figures 4B, left; Figure S4B). Four splicing-related proteins also showed reduced binding to Z18^trunc^ (FNBP4, PRPF4B, RBM25, and PRPF40A) (Figure S4C). In contrast, PABPC1 and SRRT had stable or increased binding to Z18^trunc^ (Figures 4B, right; Figure S4D). PABPC1 is a polyadenosine-binding protein that is often amplified in cancer^39^ and can protect RNAs from decay.^40^ Although PABPC1 is essential for LINE-1 retrotransposition,^41^ it has no reported role in NEXT. In contrast, SRRT is a cap-binding protein that associates with NEXT.^42^ No additional cap-binding proteins were detected above background (NCBP1 [CBP80], NCBP2 [CBP20], NCBP3, PHAX, or eIF4E). Thus, Z18^trunc^ loses NEXT binding but retains PABPC1 and SRRT, consistent with direct ERV RNA stabilization.

To consider how Z18^trunc^ exerts its dominant-negative activity, we proposed two models: Z18^trunc^ could bind and sequester (1) Z18^WT^ from its RNA substrates (Figure S4E, model 1) or (2) substrate RNAs away from Z18^WT^ (Figure S4E, model 2). In model 1, Z18^trunc^ should be predominantly localized in the same compartment as Z18^fl^ and should efficiently bind Z18^fl^. We previously noticed by IP-MS that Z18^trunc^ was found at lower levels in the nuclear fraction than Z18^fl^ (Figure S4F). We hypothesized that Z18^trunc^ was being exported to the cytoplasm, as was previously seen for a C-terminal mutant of Z18.^23^ V5-tagged Z18 immunoblots indicated that Z18^fl^ was enriched in the nucleus as expected, while Z18^trunc^ was enriched in the cytoplasm (Figure S4G) where it continued to associate with SRRT and PABPC1 (Figure S4H). Z18 immunofluorescence (IF) in Z18^trunc^-expressing cells confirmed Z18^trunc^ cytoplasmic localization versus control A375 cells (Figures 4C and 4D). Consistently, IF in Z18^trunc/+^ versus control Z18^WT^ lines showed a higher cytoplasmic to nuclear ratio of Z18 (Figures 4E and S4I). These data indicate that Z18^trunc^ is cytoplasm enriched.

To test whether Z18^trunc^ could sequester Z18^WT^ from its substrate, we asked whether Z18^trunc^ bound Z18^fl^. Z18 IP-MS unique peptide measurements were plotted along the protein length (Figure S4J). As expected, Z18^trunc^ samples (+/− RNase) had only a few peptides measured distal to the truncation site, suggesting that Z18^trunc^ does not pull down Z18^fl^. To confirm that Z18^trunc^ does not sequester Z18^fl^, we performed IP-western blot analyses of Z18^trunc^ from nuclear and cytoplasmic fractions to detect Z18^fl^, finding no signal (arrows in Figures S4K and S4L).

Because Z18^trunc^ is dominant negative, does not sequester Z18^WT^, and is cytoplasm enriched, we hypothesized that it sequesters ERV RNA away from the surveillance machinery. To determine the localization of Z18-regulated ERV RNA, we performed RNA *in situ* hybridization for the specific *MER4-int* transcript using BaseScope in Z18^trunc^-expressing versus control A375 cells. We found that the *MER4-int* transcript is enriched in the cytoplasm relative to the nucleus in Z18^trunc^-expressing cell lines (*n* = 2) versus control cells (Figures 4F, 4G, and S4M–S4O). This suggests that Z18^trunc^ binds and stabilizes ERV messages in the cytoplasm, sequestering them from the degradation machinery.

We next considered how Z18^trunc^ might exert its dominant-negative activity by directly binding ERV RNA (Figure S4E, model 2). Since the ability of Z18 to regulate retroelement RNA is highly evolutionarily conserved, we analyzed Z18’s homology to determine how Z18 could bind RNA. By aligning Z18 homologs from human, mouse, zebrafish, *C. elegans*, and D*. melanogaster* using a tool called MUSCLE,^43^ we found two highly conserved domains: the RNA-surveillance recruitment domain and the zinc-finger domain after which Z18 is named (zinc-finger alignment in Figure S4P). Z18 is one of 57 human proteins with a CCCH zinc-finger motif, a protein class that often binds RNA and functions in immunity.^44^ Since the Z18 structure is unknown, we used the RBM22^45^ (PDB: 6ID1) structure to create a homology model of the Z18 zinc-finger domain (Figure S4Q). Our model predicted that the CCCH zinc-finger domain has a Zn^2+^ ion coordinating with C224, C232, C238, and H242 (Figure 4H) and that the highly conserved aromatic residues F226, W234, and F240 directly bind RNA via pi-pi stacking interactions (Figure 4I). We hypothesized that Z18^trunc^ binds RNA via the aromatic amino acids in the zinc-finger domain.

To test whether Z18 directly binds ERV RNA via the aromatic amino acids in the zinc-finger domain, we used photoactivatable ribonucleoside-enhanced crosslinking and immunoprecipitation (PAR-CLIP).^46^ V5-tagged Clover, Z18^fl^, Z18^trunc^, and Z18^trunc^ zinc-finger aromatic mutant (hereafter Z18^truncZnFmut^) were transiently expressed in A375 human melanoma cells (*n* = 3 each) and IPed. Bound RNA was sequenced, and differential TE binding relative to Clover was assessed using SQuIRE (Figures 4J and S4R–S4T). Z18^fl^ significantly bound numerous TE RNAs (4,702), Z18^trunc^ maintained binding to most TE RNAs (4,134), while Z18^truncZnFmut^ bound fewer TE RNAs (1,266) (*p* < 0.05, DESeq2) (Figures 4K and S4U, example transcript in S4V, S4W, and S4X). These data show that aromatic residues in the Z18 zinc-finger domain are required for stable TE binding. Z18^trunc^ bound significantly more LINE elements than Z18^fl^, so we asked whether LINE-1 retrotransposition contributes to Z18^mut^ phenotypes. Using catalogs of published somatically acquired LINE-1 insertions in a pan-cancer study,^47^ we found no statistical increase in LINE-1 retrotransposition in Z18^mut^ cancers (average Z18^mut^ 10.09, *n* = 11; average rest 6.57, *n* = 326; *p* = 0.72, two-sided *t* test). We also found no change in LINE-1 open reading frame 1 (ORF1) protein expression in the Z18^trunc^-expressing cell lines (Figure S4Y). Thus, Z18^fl^ and Z18^trunc^ directly bind LINE and LTR/ERV RNAs, and zinc-finger aromatic residues are required for stable TE binding.

To determine the specific RNA sequences bound by Z18 in the PAR-CLIP data, we used wavClusteR,^48^ to call high-confidence T-to-C transition regions (clusters). Z18^fl^ and Z18^trunc^ had a similar number of clusters (296,229 and 284,329, respectively) while Clover yielded fewer clusters (144,336), which represented background RNA binding. To determine the identity of Z18-bound RNAs, clusters were annotated for LTRs, LINEs, introns, and exons (Figures S4Z–S4ZC). We found that Z18^fl^ and Z18^trunc^ clusters were enriched for LTRs, LINEs, and introns relative to exons (Figures 4L, 4M, and S4Z–S4ZC; Table S5, example Z18-bound transcript with intron inclusion in Figure S4ZD). In addition, Z18^trunc^ bound other NEXT targets, such as PROMPTs and eRNAs (Figures S4ZE and S4ZF), which were not regulated by Z18^trunc^ (Figure S3V). This is consistent with our findings that Z18^trunc^ preferentially regulates polyadenylated TEs (Figures S3Q versus S3U and S3W) and retains binding to PABPC1, a polyA-binding protein (Figure 4B). Together, these data indicate that Z18^trunc^ binds and regulates polyadenylated TEs, particularly ERVs.

To determine which RNA motifs are bound by Z18, a simple enrichment analysis (SEA) was performed.^49^ This analysis revealed significant enrichment of multiple motifs within a subset of sequences, suggesting that Z18 does not bind to a specific motif, consistent with published reports^50^ (Table S6). We noticed that many of the most enriched motifs were A/U rich and found that, among the top 12 (log *q* value <−10, highest true-positive %), A and U made up 83% of the single-nucleotide bases (Figure S4ZG). As introns^51^ and retroelement^52^ messages have higher AU nucleotide content than exons, and AU RNA was enriched in the top motifs, we tested whether Z18-bound regions were broadly enriched for AU RNA relative to Clover. We found that both Z18^fl^ and Z18^trunc^ bound more AU-rich RNA than Clover, and this effect was decreased for the Z18^truncZnFmut^ (Figure S4ZH). These analyses indicate that Z18^fl^ and Z18^trunc^ directly bind and regulate AU-rich polyadenylated retrotransposon RNA via conserved aromatic residues in the zinc-finger domain, supporting a dominant-negative mechanism via RNA binding.

### Z18-regulated ERV RNA is sufficient and necessary for Z18 mutation-associated melanomagenesis

**Figure 5.**
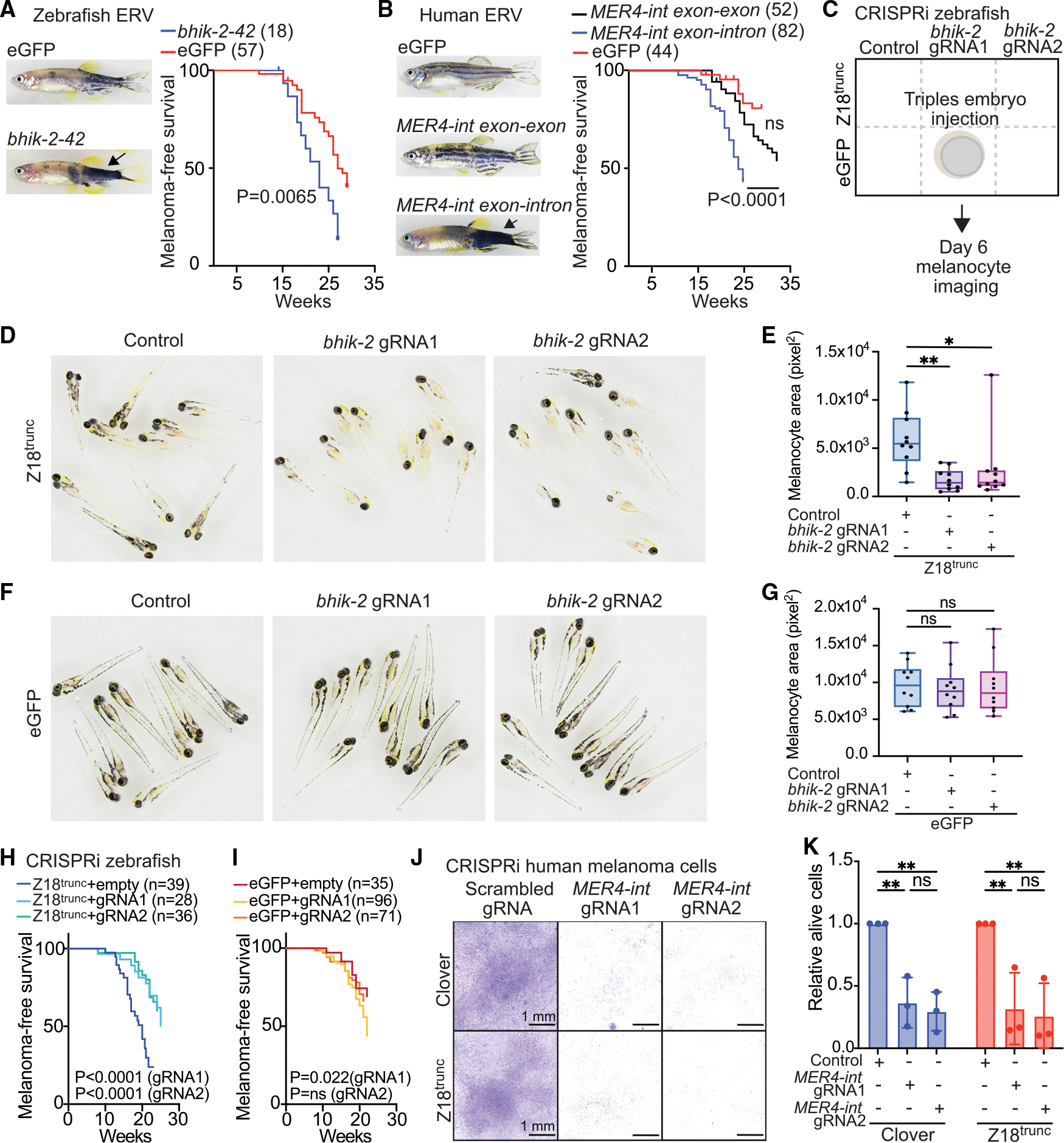
Z18-regulated ERV RNA is sufficient and necessary for Z18 mutation-associated melanomagenesis (A and B) Melanoma-free survival for triple-mutant zebrafish with melanocyte-specific expression of (A) *bhik-2–42* versus eGFP. Log rank. (B) *MER4-int* exon-exon, *MER4-int* exon-intron, or eGFP. Log rank. *n* = zebrafish. ns = non-significant. (C) Schematic of melanocyte-specific CRISPRi of *bhik-2* in eGFP or Z18^trunc^ melanocytes. (D and E) Triple-mutant zebrafish with Z18^trunc^ and CRISPRi of control or *bhik-2* (gRNA1 or gRNA2) in melanocytes at 6 dpf. (D) Light micrographs. (E) Mean melanocyte area. *n* = 10 zebrafish per genotype. *p = 0.0486, **p = 0.0018 (Welch’s *t* test). (F and G) Triple-mutant zebrafish with eGFP and CRISPRi control or *bhik-2* (gRNA1 or gRNA2) in melanocytes at 6 dpf. (F) Light micrographs. (G) Mean melanocyte area. *n* = 10 zebrafish per genotype. ns = non-significant (Welch’s *t* test). (H and I) Melanoma-free survival for melanocyte-specific CRISPRi of control or *bhik-2* (gRNA1 or 2) in (H) Z18^trunc^ or (I) eGFP. ns = non-signficant. (J and K) Crystal violet-stained viable cells from Clover (upper) and Z18^trunc^-expressing (lower) cell lines transduced with CRISPRi control gRNA or *MER4-int* gRNA1/2. (J) Light micrographs. (K) OD measurements. Clover (blue, left); MER*4-int* gRNA1 **p = 0.0039, gRNA2 **p = 0.0018. Z18^trunc^ (red, right); *MER4-int* gRNA1 **p = 0.0023, gRNA2 **p = 0.0012. ns = non-significant (two-way ANOVA). See also Figure S5.

Because Z18^trunc^ accelerates zebrafish melanoma and stabilizes ERV RNA, we tested whether ERV RNA directly contributes to oncogenesis. We expressed the two most significantly upregulated ERVs from Z18^trunc^-expressing zebrafish melanomas, chr4:42592945 *bhik*-2 (hereafter *bhik-2–42*) and chr4:37858940 *bhik*-2 (hereafter *bhik-2–37*), in the zebrafish system. Notably, expression of either ERV was sufficient to expedite melanoma onset in zebrafish (Figures 5A and S5A–S5C; Table S4). Both zebrafish ERVs are solo LTRs, i.e., they underwent recombination, resulting in loss of intervening protein-coding regions, and are therefore predicted to be noncoding. Thus, we hypothesize that *bhik-2* RNA can directly contribute to oncogenesis.

To test whether Z18-regulated ERV-driven oncogenesis is evolutionarily conserved, we expressed the two ERV RNAs that are most stabilized in Z18^trunc/+^ and Z18^trunc^-expressing human melanoma cells, *MER4-int* RNA and *HERVK3-int*. Both human ERVs have spliced and unspliced isoforms, so we tested the oncogenic potential of both transcripts. We expressed spliced *MER4-int* (*MER4-int* exon-exon) and unspliced *MER4-int* (*MER4-int* exon-intron) (Figure S5D) in the triple-mutant zebrafish melanoma model. *MER4-int* exon-intron expression produced increased black patches (early melanoma) at 10.7 weeks (Figure 5B, left; Figure S5E) and expedited melanoma onset compared to *MER4-int* exon-exon and eGFP expression (Figure 5B, right; Figures S5I and S5J). We also tested whether Z18^trunc^-regulated *HERVK3-int* would expedite melanoma (unspliced: *HERVK3-int* exon-intron; spliced: *HERVK3-int* exon-exon) (Figure S5F) and found that both transcripts caused increased black patches at 10.7 weeks (Figures S5G and S5H, left) and expedited melanoma onset compared to an eGFP-expressing control (Figures S5H, right, S5K, and S5L). These data indicate that human ERV RNA itself can contribute to oncogenesis in a conserved manner.

To test whether ERV RNA is required for Z18 mutant-driven oncogenesis in zebrafish, we developed a melanocyte-specific CRISPR inhibition (CRISPRi) approach to inhibit *bhik-2* expression. Catalytically dead Cas9 fused to a transcriptional repressor (dCas9-KRAB-MeCP2) was expressed in melanocytes using the *mitfa* promoter; gRNAs were expressed using a ubiquitous promoter, and a Mitfa minigene driven by the *mitfa* promoter enabled cell-autonomous, melanocyte-specific CRISPRi (Figure S5M). We tested the system using a previously validated gRNA targeting *tyrosinase*, a gene required for pigment production^53^ (gRNAs in Table S4). CRISPRi of *tyrosinase* significantly reduced melanin production in melanocytes at 6 days post-fertilization (dpf) in wild-type zebrafish (Figure S5N), indicating functionality of the system. To inhibit *bhik-2* expression, we designed gRNAs that are predicted to target multiple *bhik-2* elements (13/15 Z18-regulated *bhik-2* ERVs) (Figure S5O) and tested the efficiency of injected *bhik-2* gRNA and Cas9 protein in whole embryos using a T7E1 assay (Figure S5P). The two most efficient gRNAs were integrated into the melanocyte-specific CRISPRi system, and qPCR in whole embryos confirmed *bhik-2* inhibition (Figure S5Q), validating CRISPRi-mediated repression of *bhik-2*.

To test whether *bhik-2* expression is required for Z18-mediated oncogenesis, we performed melanocyte-specific *bhik-2* CRISPRi versus control in Z18^trunc^ or eGFP zebrafish (schematic of Figure 5C). In Z18^trunc^-expressing melanocytes, *bhik-2* CRISPRi significantly reduced the melanocyte area at 6 dpf (Figures 5D and 5E), while *bhik-2* CRISPRi had no effect in eGFP-expressing zebrafish (Figures 5F and 5G). We also found that *bhik-2* CRISPRi significantly slowed tumor onset in Z18^trunc^-expressing melanoma (Figure 5H), while *bhik-2* CRISPRi had a minimal effect on tumor onset in eGFP-expressing zebrafish (Figure 5I). Thus, *bhik-2* is required for melanocyte development and melanoma onset in the Z18^trunc^ context.

To test whether Z18^trunc^ human melanoma cells require ERV RNA, we performed CRISPRi of *MER4-int* in Z18^trunc^-expressing and control human melanoma cells. Two gRNAs were confirmed to inhibit *MER4-int* expression using transient transfection as compared to no transfection or a control gRNA (Figure S5R) (both *MER4-int* gRNAs, zero perfect genome matches). These two gRNAs were then introduced into Z18^trunc^- or Clover-expressing A375 human melanoma cell lines using lentivirus (*n* = 3, each). Cells were selected with antibiotics to ensure CRISPRi delivery, expanded, and stained with crystal violet to assess the cell number. *MER4-int* gRNAs significantly reduced numbers compared to a control gRNA in both Z18^trunc^ and Clover cell lines (Figures 5J, 5K, and S5S). This result shows that ERV RNA is required for A375 cancer cell growth and supports a functional oncogenic role for ERV RNA in human melanoma.

## DISCUSSION

Our findings identify Z18 mutation as a mechanism of ERV RNA accumulation in cancer and support a direct oncogenic role for ERV RNA. Although we initially expected Z18^trunc^ to affect ptRNAs, it instead caused selective accumulation of retroelement RNAs, especially ERVs, across zebrafish, patient tumors, and engineered human cells. These results broaden our prior work on oncogenic RNA surveillance defects^6^ and suggest that distinct perturbations in nuclear RNA quality control can promote cancer through accumulation of different aberrant polyadenylated RNAs.

We found that Z18 deletions are common and significantly enriched in cancer (36.6% all cancers; 30.0% melanoma). Although Z18’s role in cancer has not been investigated previously, one case report described transformation of severe congenital neutropenia into acute myeloid leukemia, with the offending clone harboring a Z18^trunc^ mutation (777fs),^54^ suggesting that although rare, truncating mutations are functional. Z18 truncating mutations are statistically enriched just upstream of the domain required to recruit nuclear RNA degradation machinery, supporting a role beyond loss of function. Consistent with this, Z18^trunc/+^ enhanced ERV RNA accumulation more than loss of one Z18 copy, demonstrating dominant-negative activity. We previously showed that CDK13 mutations also act in a dominant-negative manner,^6^ suggesting a common mechanism in nuclear RNA surveillance mutations. We hypothesize that dominant-negative mutations perturb RNA metabolism while avoiding lethality from complete loss of function.

Z18, which is named for its CCCH zinc-finger domain (ZC3H18), is one of nearly 60 human CCCH zinc-finger proteins.^44^ Where studied, CCCH zinc fingers bind RNA, and many, including Z18, function in immune responses.^44,55^ We found that Z18 normally binds ERV RNA and targets it for degradation. Because retroelements comprise nearly half of the human genome,^11,56^ even low-level transcription could plausibly affect cellular phenotypes.^12^ Multiple organisms have therefore evolved mechanisms to suppress ERV expression, even for immobile elements. The best described is transcriptional silencing by Krab zinc-finger proteins (KZFPs). The role of KZFPs in limiting TE expression is so profound that multiple studies have shown that TE evolution drives host zinc-finger evolution.^57–62^ Our work indicates that RNA-binding zinc fingers can promote ERV RNA degradation, complementing DNA-binding zinc-finger-mediated transcriptional repression.

The human silencing hub (HUSH) complex has also been shown to repress expression of intron-less retroelements.^63^ Although ZCCHC8, a component of the NEXT complex, directly interacts with HUSH,^10^ Z18 was not detected with key HUSH components in prior work^23^ or in our IP-MS experiments (except PPHLN, below our filtering threshold). Instead, we observed enrichment of splicing-related proteins and found that Z18 tends to bind intron-containing transcripts. For example, two of the most regulated ERV-containing transcripts, *MER4-int* and *HERVK3-int*, were spliced with retroelement or host sequences. Spliced ERV RNAs, which are not regulated by HUSH silencing, may therefore require NEXT targeting via Z18, potentially explaining Z18 specificity for TE targets over unspliced RNAs such as PROMPTs and eRNAs.

Retroelements, especially LTRs/ERVs,^16,17^ are upregulated in many cancers, suggesting positive selection for RNA production from TE loci. Intriguingly, proper ERV RNA levels are required during early embryogenesis, with both upregulation^64^ and downregulation^65^ causing lethality. Recently, proteins encoded by zebrafish Z18-regulated *bhik-2* elements were shown to be required for neural crest development.^66^ Because cancers often co-opt developmental programs, these studies suggest that, despite their immobility, ERV RNAs may be repurposed to promote oncogenesis. Consistent with this model, zebrafish and human Z18-target ERVs accelerated melanoma onset in zebrafish, showing that aberrant ERV expression itself can contribute to oncogenesis. Z18-regulated *bhik-2* ERV RNA was also required for Z18-driven melanoma in zebrafish, and Z18-regulated ERV expression was required for proliferation of human melanoma cells. In clear cell renal cell carcinoma, VHL loss drives HIF2-dependent ERV RNA upregulation despite immune responses to ERV antigens,^67^ suggesting that other common cancer alterations may likewise activate ERV RNA.

Our findings suggest that the Z18-ERV RNA axis may have therapeutic implications. We observed that Z18 mutations activated viral mimicry gene expression, similar to ERV de-repression,^17,36,37^ which can increase immune infiltration, antigenicity, and responses to immune therapy in multiple cancers.^17–19^ ERV-encoded antigens are increasingly recognized as targets of anti-tumor immunity.^67^ We therefore hypothesize that patients with Z18-mutant cancers may respond better to immunotherapy due to activation of innate immune signaling or recognition of translated ERV-encoded peptides. In addition, we hypothesize that transient Z18 inhibition in Z18^WT^ cancers could potentially sensitize tumors to immunotherapy. Given that ~30% of patients harbor Z18 loss or mutation and that Z18 modulation may be therapeutically tractable, this pathway is likely to be clinically relevant. More broadly, our work supports the concept that defective nuclear RNA degradation shapes the cancer transcriptome, contributes to oncogenesis, and reveals therapeutic vulnerabilities.

### Limitations of the study

Our data support an oncogenic role for ERV RNA, but the mechanism remains unknown. We also do not yet fully understand why Z18 mutation or loss selectively affects retroelements while leaving other NEXT substrates intact. In addition, we found that human cells depend on one Z18-regulated ERV RNA, although it remains unclear whether this reflects a broader dependence on ERV RNA. Finally, because the ERV RNA CRISPRi experiments were performed in a single parental human melanoma cell line, additional human cell models are needed to establish generalizability.

## RESOURCE AVAILABILITY

### Lead contact

Requests for further information and resources will be fulfilled by the lead contact, Megan L. Insco (megan_insco@dfci.harvard.edu).

### Materials availability

All zebrafish strains, human cell lines, and DNA vectors are available through the corresponding author. The zebrafish melanocyte-specific CRISPRi vector has been deposited (AddGene, 251742).

### Data and code availability

- All generated data have been deposited at GEO and are publicly available as of the data of publication. Please see the key resources table for details.
- This paper also analyzes publicly available data from GDC and GEO. Details are provided in STAR Methods.
- This paper does not report original code.
- Any additional information required to reanalyze the data reported in this paper is available from the lead contact upon request.

## STAR★METHODS

### EXPERIMENTAL MODEL DETAILS

#### Animals

Animal studies were approved by the Animal Care and Use Committee at the Dana-Farber Cancer Institute (IACUC 21–027). Three strains of zebrafish were used: wild-type AB, Casper (mitfa−/−; roy−/−),^68^ and the Triple melanoma model (*mitfa*−/−*; mitfa:BRAF*^*V600E*^*; p53*−/−).^27^ Casper fish were genotyped visually because they have a distinctive appearance resulting from the absence of most pigmentation cells. The Triple melanoma model was outcrossed to maintain strain health and then incrossed every four generations; incrossed animals were genotyped to confirm homozygosity for the three genetic alterations. The developmental stage varied by experiment and is described in the relevant sections below. Animals were fed and monitored daily for changes in health or signs of distress as specified in the animal protocol. Water parameters were continuously monitored. Animals exhibiting signs of distress or tumors exceeding 10% of adult zebrafish body weight were isolated and euthanized in ice water. Matching animal inclusion by sex was not feasible, as sex is environmentally determined and not morphologically apparent until 3 months of age. Age and sex were matched for tumor collection where possible.

#### Cell lines

A375 human melanoma cells (ATCC CRL-1619) or SW480 colon adenocarcinoma cells (ATCC CCL-228) were identity-verified via short tandem repeat analysis, then used for transient transfections or stable line generation. Routine monthly mycoplasma testing was performed (Sigma, MP0025). All cell lines were mycoplasma negative. Cells were grown at 37°C in DMEM with 4.5 g/L D-glucose and L-glutamine supplementation with 10% FBS and penicillin/streptomycin or selection antibiotics as needed. A375s are derived from a female and SW480 cells are derived from a male; we do not expect sex to affect results, as Z18-mutant-derived ERV expression occurs in both male and female patients.

### METHOD DETAILS

#### Mutational analysis of patient samples

We downloaded cutaneous melanoma datasets consisting of 1,347 samples from cBioPortal^39^ and the International Cancer Genome Consortium (ICGC) Data Portal (Data S1, “Z18 Mut & Melanoma Patient Refs”) to identify all Z18 mutations. Mutations that matched germline mutations were excluded. The PanCancer dataset^20^ was obtained from The Cancer Genome Atlas (TCGA) and underwent PolyPhen-2^21^ analysis (Data S1, “PolyPhen-2 Results”). GISTIC statistics were gathered from tumorscape.org via the “Gene-Centric” analysis.^22^ Next, a dataset of nonredundant studies spanning various cancer types from cBioPortal was compiled. In total, 85 unique studies were identified (Data S1, “Pan-cancer Patient Sample Refs”), encompassing 23,411 samples, each containing at least one sample with a mutation in the *Z18* gene. Duplicate or serial samples from the same individual were excluded. Amino acid residues with >5 mutations were plotted. To analyze whether truncating mutations are enriched upstream of the RNA surveillance recruitment domain, a Fisher’s exact test was used (Data S1, “Z18^trunc^ Fisher’s Exact Test”). MSIsensor^83^ scores were used to identify microsatellite-unstable samples within the TCGA PanCancer dataset.^20^ Samples with Z18 truncating and nontruncating mutations were compared to Z18 wild-type (Z18^WT^) samples to determine which were associated with microsatellite instability (MSIsensor >10) using an odds ratio and a Fisher’s exact test (two-sided) (Table S1).

#### TCGA Z18 mutant repeat element expression analysis

##### Melanoma (SKCM) sample selection

Samples with deleterious Z18 missense (AlphaMissense >0.564), nonsense, or frameshift mutations with publicly available RNA-seq and clinical information were downloaded on 4/30/25 (TCGA Firehouse Legacy and DFCI 2019)^20,84^ (*n* = 10). Age, sex, stage, and driver mutations (BRAF [V600 or type II BRAF], NRAS Q61, and NF1) were also downloaded. Z18^WT^ samples (*n* = 10) were selected to match the Z18^mut^ samples by study, stage, sex, and driver mutation and were then downloaded. Age was only available for TCGA samples and was matched accordingly. See also Data S2.

##### Uterine cancer (UCEC) sample selection

Samples with Z18 missense (AlphaMissense >0.564), nonsense, or frameshift mutations with publicly available RNA-seq and clinical information were downloaded on 8/10/25^20^ (*n* = 14). Samples with Z18 hotspot truncations (amino acid 679–901) were prioritized. Sex, age, and driver mutations that are reported to affect >20% of patients, as well as the three most frequently mutated mismatch repair genes were downloaded (PTEN, PIK3CA, CTNNB1, ARID1A, KRAS, TP53, PIK3R1, ZC3H18, MLH1, MSH2, MSH6). Z18^WT^ samples (*n* = 14) were selected to match the Z18^mut^ samples by sex and driver mutation and were then downloaded. Stage was not considered, as staging information was not available. See also Data S2.

##### Colon cancer (COAD) sample selection

Samples with Z18 missense (AlphaMissense >0.564), nonsense, or frameshift mutations with publicly available RNA-seq and clinical information were downloaded on 8/10/25^20^ (*n* = 11). Samples with Z18 hotspot truncations (aa 679–901) were prioritized. Stage, sex, age, and driver mutations that are reported to affect >20% of patients, as well as the three most frequently mutated mismatch repair genes were downloaded (KRAS, APC, TP53, PIK3CA, ZC3H18, MLH1, MSH2, MSH6). Z18^WT^ samples (*n* = 11) were selected to match the Z18^mut^ samples by stage, sex, age, and driver mutations, and were then downloaded. See also Data S2.

##### Analysis

BAM files pertaining to the above samples were converted to FASTQ prior to processing with SQuIRE. LTR/ERV loci-specific FPKM values were collected. All “NA” FPKM values were replaced with 0s (to account for no detected TE expression) and only TE loci expressed in at least one sample on the same strand as the TE locus were included. Wilcoxon tests were used to compare expression of individual LTR/ERV locus in mutant versus wild-type samples and significantly changed loci (*p* < 0.05) were plotted for all three TCGA datasets.

#### Zebrafish experiments

##### Cloning

The MiniCoopR (MCR) Gateway system was used.^27^ MCR overexpression constructs were assembled using Gateway vectors including the *mitfa* promoter, relevant coding sequence, 3′ polyA tail, and *MCR* destination vector with LR Clonase II Plus (Thermofisher, 12538120). The human Z18 coding sequence was obtained from Dharmacon (Accession: MHS6278–202759301 Clone ID: 6042036). Truncated Z18 was cloned with the R680Gfs*5 (hereafter Z18^trunc^) patient mutation using *in vitro* mutagenesis (NEB, E0554S). Gene fragments for the chr4:42592945 *BHIKHARI-2* ERV (hereafter *bhik-2–42*) and chr4:37858940 *BHIKHARI-2* ERV (hereafter *bhik-2–37*) were ordered from BioTwist, and each fragment was assembled into SalI and EcoRI linearized MCR vector (NEB, E2621). The *MER4-int* (exon-exon and exon-intron) and *HERVK3-int* (exon-exon and exon-intron) constructs were synthesized into the MCR MCS vector by GenScript.

##### Melanoma model

ARRIVE guidelines were followed where possible.^85^ Experiments were performed as published.^27,31^ Briefly, *p53*^−/−^*; mitfa:BRAF*^*V600E*^*; mitfa*^−/−^ (triple-mutant) one-cell embryos were injected with either 20 ng/μL of control or experimental DNA along with *in vitro*-transcribed Tol2 RNA for integration.^27^ In all experiments, DNA that overexpressed the gene of interest was marked with a *mitfa* minigene to rescue Mitfa, allowing cell-autonomous melanocyte genetics (schematic in Figure S1A). Control vectors expressed eGFP. When multiple vectors were injected, total DNA was 20 ng/μL, split equally among vectors. Embryos were sorted for melanocyte rescue (vector integration) at 6 days post-fertilization (dpf). Equal numbers of zebrafish were raised in control and experimental tanks to control for density effects. Zebrafish with >10% of body length showing melanocyte rescue were anesthetized in tricaine and imaged with the Nikon SMZ18 stereo microscope at 9 weeks (for Z18^trunc^) or 10.7 weeks (for ERV-expressing). Black patches that disrupted normal stripes and had a diameter greater than the distance between that zebrafish’s eyes were quantified from fish images. Zebrafish were scored for emergence of raised melanoma lesions ≥1 mm^2^ in subsequent weeks. Melanoma-free survival curves and Log rank tests were generated in Prism.

##### Melanocyte-specific CRISPRi

Zebrafish codon-optimized dCas9-KRAB-MeCP2 (CRISPRi) sequence was provided by the McCallion lab^53^ and synthesized into the MCR expression vector by GenScript. Melanocyte-specific CRISPRi function was validated using a gRNA targeting the *tyrosinase* promoter^53^ in wild-type zebrafish (using Tol2 RNA for integration). Images to assess melanocyte pigment intensity were then acquired at 6 dpf. gRNAs 1 and 2 targeting *bhik-2* were selected via CRISPOR^74^ and Integrated DNA Technologies (IDT), then cloned into the CRISPRi MCR vector. Both selected gRNAs were predicted to target 13 of the15 top Z18-regulated *bhik-2* elements. gRNA efficiency was assessed by injecting 10ng of *in vitro*-transcribed *bhik-2* gRNA +200ng of Cas9 protein (PNA Bio, CP01) into *mitfa*^−/−^*; roy*^−/−^ (Casper) embryos.^68^ Genomic DNA was collected at 1 day post-fertilization (dpf), PCR-amplified across the *bhik-2–37* gRNA cut site, and analyzed by T7E1 assay. Both gRNAs cut efficiently and were used in subsequent experiments; a no-gRNA CRISPRi vector served as the control. Melanocyte-specific CRISPRi gRNA efficiency was tested at 3 dpf using *bhik-2–42* qPCR in whole triple-mutant embryos. In triple-mutant embryos, CRISPRi control, *bhik-2* gRNA1, or gRNA2 were co-injected with either eGFP or Z18^trunc^ vector, and melanocyte area was quantified at 6 dpf using ImageJ ROI Manager. Melanoma scoring, plotting, and analyses were as described above. Primers in Table S4.

##### Western blotting

Protein was quantified using the DC Protein Assay (Bio-Rad, 5000116). The following antibodies were used: anti-Z18 (Novus, NBP1–82570, 1:1000), anti-beta actin (CST, 3700S, 1:1000), anti-GFP (Santa Cruz, sc-9996, 1:1000). Rabbit or mouse secondary HRP antibodies were incubated for 1 h at room temperature (20°C) (CST, 7074S or 7076S, 1:2000). Films were developed with Pierce ECL substrate (Thermofisher, 32106).

##### RNA-seq

Triple-mutant zebrafish melanomas from different individual fish with expression of eGFP (*n* = 3) or Z18^trunc^ (*n* = 3) were collected. Tumors were homogenized, RNA was purified (Zymo Research, R1054), DNA was removed, and RNA was polyA-selected (NEB, E7490S). Additional eGFP (*n* = 3) or Z18^trunc^ (*n* = 3) melanomas were processed similarly and rRNA-depleted (NEB, E7405). All samples underwent stranded library preparation (NEB, E7760S) and were sequenced on a HiSeq 4000.

##### RNA-seq custom genome mapping to verify transgenes

The raw sequence reads from polyA-selected and ribo-minus RNA-seq were trimmed using Cutadapt (v. 4.6) with the parameters: cutadapt -j 4 -q 30 -a GATCGGAAGAGCACACGTCTGAACTCCAGTCAC -a CTGTCTCTTATACACATCT -g AGATGTGTATAAGAGACAG -A GATCGGAAGAGCGTCGTGTAGGGAAAGAGTGTAGATCTCGGTGGTCGCCGTATCATT -A CTGTCTCTTATACACATCT -G AGATGTGTATAAGAGACAG -m 50 -e 0.02.^86^ The trimmed reads were mapped to a custom reference genome containing the chromosomes of the *Danio rerio* genome build danRer11 plus the human gene *Z18*. Mapping was performed using STAR (v. 2.5.3a) in paired-end mode with parameters –outSAMtype BAM SortedByCoordinate –quantMode GeneCounts –outWigStrand Stranded –outWigType bedGraph.^80^ bedGraph files were converted to bigWig format using bedGraphToBigWig.^70^ bigWig files were visualized in Integrative Genomics Viewer (IGV).^77^

##### RNA-seq transposable elements (TE) analysis

TEs were measured from polyA-selected RNA-seq with SQuIRE^28^, which uses stringent mapping coupled with an expectation maximization-based algorithm. Reads were mapped to the danRer11 genome, counted, and examined for differential expression of individual TE loci as annotated by the RepeatMasker GTF (downloaded from UCSC on 9/20/2022). Average expression of each TE locus was calculated for all samples. Only loci with expression on the correct strand in at least one sample were considered. In cases where an individual locus was expressed in a subset of samples, “NaN” values were updated to “0”. A Wilcoxon signed-rank test with continuity correction was used to determine statistical differences in TE expression between Z18^trunc^ versus eGFP conditions. Mean LTR/ERV loci expression (FPKM) was visualized in Figure 2A. Statistics for other TE classes are included in Table S2. The average FPKM log2 fold change was plotted by the difference for all loci in Figure 2B. Uniquely mapping reads for the most highly expressed TEs with a significant change were visualized in IGV.^29^

##### RNA-seq analysis of canonical NEXT targets

Ribo-minus RNA-seq reads from eGFP or Z18^trunc^-expressing zebrafish melanomas were mapped to the *danRer*11 genome build using STAR 2.5.3a^80^ with the command parameters: STAR –runMode alignReads –twopassMode Basic –outFilterMultimapNmax 20 –outFilterMismatchNmax 999 –outFIlterMismatchNoverLmax 0.033 –alignIntronMin 70 –alignIntronMax 500000 –alignMatesGapMax 500000 –alignSJoverhangMin 8 –alignSJDBoverhangMin 1 –outSAMstrandField intronMotif –outFilterType BySJout. Bed files containing the genomic coordinates of known NEXT target RNAs were generated as follows. Enhancer regions were identified using a published H3K27ac ChIP-seq bed file from a zebrafish melanoma cell line (GEO: GSM1953851_CKK2_11_peaks.narrowPeak.gz).^31^ As this was a narrow peak file mapped to Zv9, liftOver from UCSC^78^ was used to convert Zv9 coordinates to danRer11. Using the intersectBed –v function from Bedtools version 2.30.0,^71^ H3K27ac-enriched regions that excluded gene bodies were identified as intergenic enhancers, while H3K27ac-enriched regions that overlapped the gene body excluding the 200 nucleotides downstream of the transcription site (to eliminate promoters) were identified as intragenic enhancers. PROMPTs were identified as the 200 nucleotides upstream in the antisense direction of all annotated gene transcription start sites. The mapped reads falling within each region of the NEXT target bed files were counted using the featureCounts function of Subread version 2.0.3.^76^ Counts were input into DESeq2^33^ for eGFP- versus Z18^trunc^-expressing samples.

##### RNA-seq analysis of ptRNA targets

We obtained the ptRNA genome annotation file^6^ and evaluated differential exon and ptRNA expression using DEXSeq.^75^

#### Human cell line experiments

##### CCLE TE RNA-seq analysis

Eleven cell lines from the Cancer Cell Line Encyclopedia (CCLE)^87^ with Z18 mutations with available RNA-seq data were downloaded corresponding to 4 endometrial/uterine cancer lines, 3 colon/colorectal cancer lines, 2 lung cancer lines, 1 gastric cancer line, and one lymphoma line (Data S2, “CCLE cell lines”) (SRA: PRJNA523380). As controls, we selected 11 samples without Z18 alterations, matched as closely as possible by tumor type, sex, and genetic drivers (putative driver mutations, Deep Deletions, and homdel_rec mutations from OncoPrint downloaded from cBioPortal on 08/02/25). SQuIRE^28^ was run on FASTQ files. As these data are unstranded, strandedness was not considered. TE loci that were expressed in at least one sample were included in downstream analysis. “NA” values were replaced with 0s. Significantly changed TEs between Z18^mut^ versus Z18^WT^ were included (*p* < 0.05, paired Wilcoxon test). Average TE loci FPKM expression was computed for Z18^WT^ and Z18^mut^ samples, and log2 fold changes were calculated using a 0.01 pseudocount, stratified by TE class (Figure 2D). SQuIRE call (DESeq2) was used to compare Z18^mut^ and Z18^WT^ cell lines (Figure 2E).

##### Human cell line genetic model generation

*Z18 allelic series cell lines.* gRNAs were selected via CHOPCHOP14^73^ and cloned into Gecko2.0 vectors (Addgene, 49535) which express both Cas9 and the relevant gRNA in one plasmid (Table S4). gRNA efficiency was assessed via bulk transient transfection (Invitrogen, 100022050) in A375 cells, 72-h puromycin selection, and Z18 immunoblotting. The most efficient gRNAs (>50% protein reduction) were selected for single-cell cloning using limiting dilution. To introduce the Z18^trunc/+^ mutation, we used CRISPR-mediated homology-directed repair by co-transfecting a vector expressing a sgRNA targeting within 20bp of the 680 mutation site and a single-stranded HDR donor DNA template (Integrated DNA Technologies). Z18^trunc/+^ single-cell clones were screened by Sanger sequencing, and expression of the truncated protein isoform was confirmed by immunoblotting. Heterozygous loss-of-function (Z18^−/+^) clones were selected that had (1) Sanger sequencing consistent with loss of one Z18 allele and (2) ~50% protein reduction by immunoblot. Wild-type Z18 (Z18^WT^) control cell lines were generated from cells transfected with Cas9-expressing vectors with no gRNA. These lines were also single-cell cloned, and intact Z18 was confirmed by Sanger sequencing and immunoblotting.

*Bulk Z18 CRISPR.* Since Z18 null clones were not isolated as expected (Z18 is a common essential gene in DepMap, https://depmap.org/portal^88^), transient bulk CRISPR was performed using the most efficient guide (gRNA 1, Figure S3A; Table S4) in triplicate. Cells transfected with a Cas9-expressing vector without a gRNA served as controls. Transfected cells were selected with puromycin for 48 h, and Z18 protein reduction was confirmed by immunoblot.

*Z18*^*trunc*^*-expressing lines.* pLENTI CMV Clover (fluorescent control) and pLENTI CMV Z18^trunc^-expressing human melanoma A375 or SW480 colon cancer cell lines were generated via lentiviral transduction and stable puromycin selection. Lines were made in biological triplicate and maintained in selection antibiotics.

##### RNA-seq

RNA was isolated, and genomic DNA was removed from Z18^WT^ (*n* = 2), Z18^trunc/+^ (*n* = 3), and Z18^−/+^ (*n* = 3) A375 single-cell clones; bulk-CRISPR control (*n* = 3) and Z18 CRISPR A375 cells (*n* = 3); Clover-expressing (*n* = 3) and Z18^trunc^-expressing (*n* = 3) A375 cells; and Clover-expressing (*n* = 3) and Z18^trunc^-expressing (*n* = 3) SW480 cells as described above for zebrafish. Samples were either polyA-selected (NEB, E7490) or rRNA-depleted (NEB, E7405) prior to stranded library preparation (NEB, E7760S). Samples were sequenced on a HiSeq 4000 with paired-end sequencing.

##### RNA-seq TE quantification

SQuIRE^28^ was run on FASTQ files as described above for zebrafish TE quantification, except that data were mapped to hg38. Log2 fold changes in TE FPKM for Z18^trunc/+^ versus Z18^WT^ that were identified as significant (*p* < 0.05) using the DESeq2^33^ (SQuIRE) were plotted by TE class (Figure 3D) and by *p*-value (Figure 3E). GSEA^34^ was performed using the RefSeq DESeq2^33^ output from SQuIRE. FPKM counts on the same strand as LTR/ERV loci that were expressed in at least one sample were included in downstream analysis. “NA” values were then replaced with 0s. Significantly changed TEs in Z18^mut^ versus Z18^WT^ were filtered (*p* < 0.05, Wilcoxon test). Z18^mut^ and Z18^WT^ average FPKM values were plotted. A Wilcoxon rank-sum test was used to assess changes between conditions.

##### RNA-seq analysis of canonical NEXT targets

Coordinates for promoter-upstream transcripts (PROMPTs), intergenic eRNAs, and intragenic eRNAs were defined using the GENCODE v47 human annotation and H3K27ac ChIP-seq data (ENCODE: ENCSR707BXM). Significant H3K27ac peaks were identified using MACS2. PROMPTs were defined as the antisense 200 bp regions upstream of transcription start sites (TSSs). Intergenic eRNAs were defined as H3K27ac peaks located outside of gene bodies and their 200 bp upstream promoter region. Intragenic eRNAs were defined as H3K27ac peaks within gene bodies but outside of exons and the 200 bp region downstream of the TSS. Read counts for these regions were quantified using featureCounts. Differential expression analysis was then performed using the DESeq2.

##### RNA-seq analysis of ptRNA targets

We obtained the ptRNA genome annotation file.^6^ We evaluated differential exon and ptRNA expression using DEXSeq. For visualization, exons with log2 fold change >8 were considered outliers and excluded from plots.

##### qPCR

RNA was isolated, and genomic DNA was removed (Zymo Research, 2052). First-strand cDNA was synthesized (Takara Bio, RR036A) and used for qPCR (Biotium, 31003). RNA expression was plotted relative to *GAPDH* for human cell lines or *gapdh* for zebrafish samples and normalized to the control condition. Primers are listed in Table S4.

##### 4sU pulse-chase RNA decay measurement

Cell lines were labeled with 50 μM 4-thiouridine (Sigma Aldrich, T4509) for 2 h followed by replacement with 4sU-free media. Cells were harvested immediately (time 0) or at 2 and/or 4 h post media replacement. Total RNA was isolated using TRIzol and treated with DNase I (Thermofisher, 18068015). 10 μg of 4sU-labeled RNA was biotinylated, purified, captured by streptavidin magnetic beads (Invitrogen, 65002), eluted using 100 mM dithiothreitol (DTT), and purified by ethanol precipitation. cDNA was prepared and quantified by qPCR as above.

##### Nascent RNA qPCR

A TT-seq protocol^38^ was used with a qPCR readout. Clover- and Z18^trunc^-expressing cell lines were labeled with 500 μM 4-thiouridine (Sigma Aldrich, T4509) for 5 min (min), lysed using TRIzol, then RNA was extracted. 50 μg of total RNA was DNase I-treated (Thermofisher, 18068015) and fragmented to 1000–1200 bp fragments with a Covaris E220 ultrasonicator (50 s at 105 peak incident power). Nascent RNA was biotinylated, purified, captured by streptavidin magnetic beads (Invitrogen, 65002), eluted using 100 mM DTT, and purified by ethanol precipitation. cDNA was prepared and quantified by qPCR as above.

##### Western blotting

5–15 μg of protein was run on 10% Tris-glycine gels followed by either semi-dry or wet (30 V, overnight) transfer to PVDF or nitrocellulose membranes. Membranes were blocked with 5% skim milk in TBST. The following primary antibodies were used with a 1:1000 dilution: anti-Z18 (Novus, NBP1–82570), anti-ZCCHC8 (Proteintech, 23374–1-AP), anti-PABPC1 (abcam, ab21060), anti-SRRT (Invitrogen, PA5–31593), anti-GAPDH (CST, 2118), anti-VCL (Sigma, HPA002131), and anti-beta-actin (CST, 3700). These antibodies were used with a 1:2000 dilution: Anti-V5 (abcam, ab27671) and anti-MTR4 (abcam, ab70551). Anti-LINE1 ORF1p (EMD Millipore, clone 4H1) was used with 1:5000 dilution. Rabbit or mouse secondary antibodies were diluted 1:2000 and incubated for 1 h at room temperature (20°C) (HRP: CST 7074S or 7076S) (fluorescent: LICOR 926–32211 or 926–68070). When visualizing immunoprecipitated protein, TidyBlot HRP (Biorad, STAR209P) was used at a 1:400 dilution. Films were developed with ECL substrate (Thermofisher, 32106 or Cytivia, RPN2232).

##### IP-MS

Coding sequences of full-length Z18 (Z18^fl^) and Z18^trunc^ (R680Gfs*5) (as described above in Zebrafish cloning) were assembled using the Gateway cloning system into pcDNA3.2 C-terminal V5 tag destination vectors (Thermo Fisher, 12489019). Each construct was transiently transfected into A375 human melanoma cells in triplicate (Thermo Fisher, L3000008). After 48 h, nuclei were isolated and lysed (Thermo Fisher, 78833). Anti-V5 (V5–10, Sigma, V8012) was conjugated to protein G beads (Thermo Fisher, 10004D) and used to IP V5-tagged proteins in the presence or absence of RNase A/T1 (100 μg/mL, Thermo Fisher, EN0551). IPed proteins were eluted by boiling and submitted for mass spectrometry at the Taplin Mass Spectrometry Facility at Harvard University. Data were filtered for proteins with >4 total peptides in all replicates in the most permissive condition (Z18^fl^ -RNase) and >4.5 average peptide count relative to a previous control IP (Clover-V5)^6^ (*n* = 240 proteins). The % of total peptides relative to bait Z18 was calculated. To identify significant changes in direct interactors, a two-sided *t* test was performed between +RNase Z18^fl^ and Z18^trunc^ conditions. Proteins that were significantly changed between +RNase Z18^fl^ and Z18^trunc^ (*p* < 0.05) and had an average > 5% of bait in at least one +RNase condition were plotted in GraphPad Prism (*n* = 8).

##### Immunofluorescence

Cells were seeded and grown for 24 h in chamber slides (Thermo Scientific, 154917PK) to ~50% confluence, rinsed with 1x PBS, fixed with 4% paraformaldehyde for 15 min at room temperature, washed three times with 1x PBS, permeabilized with 1x PBS with 0.1% Triton X-100 for 15 min, and blocked with 1x PBS containing 10% normal goat serum for 1 h. Cells were incubated with anti-Z18 (Novus, NBP1–82570) at 1:400 overnight at 4°C. Cells were then washed 3x in 1x PBS, incubated with Alexa 488-conjugated anti-rabbit secondary antibody (Invitrogen, A11008) at 1:2000 for 1 h at room temperature in the dark. Cells were washed in 1x PBS, counterstained with DAPI, and mounted. Images were acquired using a Nikon ECLIPSE TS2 inverted microscope with identical settings for all conditions. Raw images were imported into Fiji for quantification.

##### BaseScope RNA *in situ* hybridization

BaseScope was performed using the BaseScope Detection Reagent Kit-RED user manual provided by Advanced Cell Diagnostics (ACD). Cells were seeded and grown for 24 h in chamber slides (Thermo Scientific, 154917PK) to ~50% confluence, washed once with 1x PBS, fixed with 10% neutral-buffered formalin for 30 min, and then pretreated with protease III for 30 min at 40°C as supplied by ACD. Slides were washed twice with 1x PBS, incubated with *MER4-int* probe (from ACD) for 2 h, and then incubated sequentially with AMP1–8 reagents at 40°C in a HybEZ oven. Slides were incubated with Fast Red for 10 min at room temperature in the dark, stained with DAPI, mounted, and images were acquired with a Zeiss LSM 980 confocal microscope with 0.98 μm intervals. We used a Cy3 filter for fluorescent detection of Fast Red as advised by ACD. Identical acquisition settings were applied for all images. Z-stacks were orthogonally projected and then quantified using Fiji.

##### Z18 zinc-finger domain structural modeling

The protein sequence for Z18 was downloaded (UniProt: Q86VM9) and the zinc-finger domain spanning residues 219 to 245 was selected for homology modeling. The Z18 zinc finger three-dimensional structure was modeled using the online Swiss-Model server^82^ with the RBM22 protein from the human spliceosome cryo-EM structure with RNA oligomer (PDB: 6ID1^45^) as a template.

##### Modified PAR-CLIP

The aromatic zinc finger (ZnF) mutations (F226A, F227A, and W234A) were introduced into Z18^trunc^ using *in vitro* mutagenesis (NEB, E0554S). A prior study of the CCCH domain in the structurally similar ZC3H12 protein identified the aromatic residue equivalent to F240 to be unimportant for RNA binding; thus, we left F240 intact.^89^ We were uncertain whether F226 or F227 was important because they are both conserved, so we mutated both. V5-tagged Clover (control), Z18^fl^, Z18^trunc^, and Z18^trunc^ ZnF aromatic mutant (hereafter Z18^truncZnFmut^) constructs were transiently transfected into A375 melanoma cells (Thermo Fisher, L3000008). PAR-CLIP was performed as published^46^ except that protein size selection was not completed. At 24 h post-transfection, cells were labeled with 100μM 4sU for 16 h, media was removed, and cells were covered with PBS for UV crosslinking (2 min, 316 nm, Spectrolinker XL-1500). The crosslinked cell pellet was lysed with RIPA buffer (50 mg cell pellet/mL RIPA buffer), vortexed, and incubated on ice for 20 min before brief sonication (Covaris E220 ultrasonicator, 1 min, PIP 140.0, Duty factor 7.0, CPB 300). Protein G Dynabeads (100 μL/IP, Invitrogen, 10004D) were conjugated to anti-V5 (20 μg/IP, Sigma, V8012). IP was done in triplicate overnight at 4°C. Beads were washed once with RIPA buffer and twice with IP wash buffer (150 mM NaCl, 50 mM Tris). On-bead Proteinase K digestion (10 μL/IP, NEB, P8107S) was performed at 50 C for 45 min with gentle mixing every 5 min. RNA was isolated via acid phenol-chloroform extraction, purified using NEBNext RNA sample purification beads (NEB, E6351S), rRNA-depleted (NEB, E7405), and prepared for stranded Illumina sequencing (NEB, E7760S).

##### PAR-CLIP data analysis

Reads were trimmed and quantified using SQuIRE^28^ as above. TEs that were significantly bound to bait protein (Z18^fl^, Z18^trunc^, or Z18^truncZnFmut^) versus control Clover were identified using DESeq2.^33^

###### Identification of bound regions.

Trimmed reads were mapped to the human genome build hg38 using Bowtie (v 1.1.2) in paired-end mode with parameters: -v 2 -m 10 –best –strata.^72^ To identify high-confidence clusters of T-to-C transitions resulting from UV crosslinking of 4sU-labeled RNA to Z18, wavClusteR version 2.36.0^48^ was used. To identify a unified set of regions of interest for each condition, the wavClusteR output “cluster.bed” files were merged for replicates of each condition (Clover, Z18^fl^, Z18^trunc^, or Z18^truncZnFmut^).

###### Cluster annotation.

The combined cluster files for each condition were annotated using the bedtools intersect -c option, for: (a) LTRs and LINEs using UCSC RepeatMasker (downloaded 11/08/2023), (b) exons from refGene.gtf (downloaded 11/08/2023), and (c) introns. Introns were annotated using a.bed file including all disjoint segments of canonical introns^90,91^ using hg38.ncbiRefSeq.gtf^92^ (entries referring to patches or haplotypes were removed). The number of clusters annotated as overlapping with LTRs, LINEs, introns, or exons was calculated and plotted for each condition. The average cluster size (base pairs) for each condition was similar (Z18^fl^ 137.51; Z18^trunc^ 132.06; Clover 129.55).

###### Quantification of bound regions.

Trimmed reads were remapped to the human genome build hg38 using the splice-aware mapper STAR (v. 2.5.3a) in paired-end mode with parameters –outSAMtype BAM SortedByCoordinate –outWigStrand Stranded –outWigType bedGraph.^80^ To identify reads that overlapped Z18^fl^-bound regions from wavClusteR, each BAM was intersected with the merged Z18^fl^ using bedtools -intersect. StringTie v. 1.3.3b^81^ was used to count expression from intersected BAM files annotated for: (a) LTRs and LINEs, (b) exons, (c) introns, (d) PROMPTs, or (e) eRNAs. Normalized reads per transcript were used as input for DESeq2^33^ using a size factor of 1, comparing Z18^fl^ or Z18^trunc^ versus Clover (control). The log2 fold change of Z18^fl^ or Z18^trunc^ versus Clover for LTRs, LINEs, introns, or exons was plotted, and a nonparametric two-sided Wilcoxon rank-sum test was used to compare clusters annotated to contain LTRs, LINEs, or introns versus exons.

###### Motif analysis.

Simple enrichment analysis (SEA)^49^ was performed using the web server at https://meme-suite.org on merged wavClusteR^48^ PAR-CLIP Z18^fl^-bound regions (*n* = 3) with merged Clover (*n* = 3) as background and the human HOCOMOCO v11 motif database.^93^ Motifs identified by SEA were filtered to retain those with the highest true-positive rate and log10 q-value <−10. The top 12 motifs were selected, and nucleotide composition was calculated.

###### Nucleotide base composition analysis.

The genomic coordinates for each cluster.bed file were used to calculate the coverage in the corresponding BAM files using mpileup from SAMtools.^79^ Then the cluster nucleotide composition was calculated.

##### CRISPRi

We used the CRISPRi pXPR_066 all-in-one plasmid, which expresses dCas9-KRAB and gRNA sequences in the same vector, obtained from the Genetic Perturbation Platform (GPP, Broad Institute).^69^ gRNAs were designed to target the most highly regulated ERV RNA, chr14 *MER4-int* using CRISPOR,^74^ and a control gRNA targeting an intergenic site used by GPP as a control. Control gRNA and *MER4-int* gRNA1 or gRNA2 were cloned into the above vector, and gRNA efficiency was assessed via bulk transient transfection (Lipofectamine 3000, Invitrogen, 100022050). Z18^trunc^-expressing A375 cells were transfected, grown for 36 h, then selected with puromycin for 48 h. RNA was isolated, and qPCR for *MER4-int* was performed. After confirming gRNA efficacy, the three gRNA constructs were used to generate lentivirus, which was transduced into independently derived Clover- and Z18^trunc^-expressing A375s (*n* = 3 each). After 48 h, cells were selected with puromycin for 72 h, grown for 4 days without antibiotics, washed with 1x PBS, fixed with 10% formaldehyde for 10 min, stained with crystal violet for 30 min at RT, rinsed with water, and dried overnight. Images were acquired using a Nikon SMZ18 stereo microscope. Crystal violet was solubilized in methanol, and optical density was measured at 570 nm with a CLARIOstar plate reader. Primers in Table S4.

### QUANTIFICATION AND STATISTICAL ANALYSIS

The statistical details for all analyses are included in either the methods or in the relevant figure legends, including the statistical test used, the value of *n,* what *n* represents, and data dispersion measures.

## Supplementary Material

1

Data S1

Data S2

Data S3

Data S4

SUPPLEMENTAL INFORMATION

Supplemental information can be found online at https://doi.org/10.1016/j.celrep.2026.117526.

## Figures and Tables

**KEY RESOURCES TABLE:** 

REAGENT or RESOURCE	SOURCE	IDENTIFIER

Antibodies		

Anti-beta-Actin (8H10D10)	Cell Signaling Technology	Cat# 3700S; RRID: AB_2242334
Anti-GAPDH	Cell Signaling Technology	Cat# 2118; RRID: AB_561053
Anti-GFP	Santa Cruz Biotechnology	Cat# sc-9996; RRID: AB_627695
Anti-LINE1 ORF1p	Sigma-Aldrich	Cat# MABC1152; RRID: AB_2941775
Anti-MTR4 (SKIV2L2)	Abcam	Cat# ab70551; RRID: AB_1270701
Anti-PABPC1	Abcam	Cat# ab21060; RRID: AB_777008
Anti-SRRT	Invitrogen	Cat# PA5-31593; RRID: AB_2549066
Anti-V5	Abcam	Cat# ab27671; RRID: AB_471093
Anti-V5 (V5-10)	Sigma-Aldrich	Cat# V8012; RRID: AB_261888
Anti-VCL	Sigma-Aldrich	Cat# HPA002131; RRID: AB_1080570
Anti-ZC3H18	Novus	Cat# NBP1-82570; RRID: AB_11014880
Anti-ZCCHC8	Proteintech	Cat# 23374-1-AP; RRID: AB_2879269
Alexa Fluor^™^ 488	Invitrogen	Cat# A11008; RRID: AB_143165
Mouse secondary HRP	Cell Signaling Technology	Cat# 7076S; RRID: AB_330924
Rabbit secondary HRP	Cell Signaling Technology	Cat# 7074S; RRID: AB_2099233

Bacterial and virus strains		

E. coli Stbl3	NEB	Cat# C3040
E. coli DH5-α	NEB	Cat# C2987

Biological samples		

Zebrafish embryos and melanomas from variety of genotypes	This paper	NA

Chemicals, peptides, and recombinant proteins		

4-Thiouridine	Sigma Aldrich	Cat# T4509
Amersham ECL Prime Western Blotting Detection Reagent	Cytivia	Cat# RPN2232
Cas9 Protein	PNA Bio	Cat# CP01
DNase I	Thermo Fisher Scientific	Cat# 18068015
Dynabeads Protein G Beads	Thermo Fisher Scientific	Cat# 10004D
Dynabeads Streptavidin Magnetic Beads	Invitrogen	Cat# 65002
Fast EvaGreen Master Mix for qPCR	Biotium	Cat# 31003
Lipofectamine 3000	Thermo Fisher Scientific	Cat# 100022050
NEBuilder HiFi DNA Assembly Master Mix	NEB	Cat# E2621
Pierce ECL Western Blotting Substrate	Thermo Fisher Scientific	Cat# 32106
PrimeScript RT Master Mix	Takara Bio	Cat# RR036A
Proteinase K	NEB	Cat# P8107S
QIAzol lysis reagent	Qiagen	Cat# 79306
RNase A/T1	Thermo Fisher Scientific	Cat# EN0551
TidyBlot HRP	Biorad	Cat# STAR209P

Critical commercial assays		

BaseScope Detection Reagent Kit-RED	Advanced Cell Diagnostics (ACD)	NA
LR Clonase II Plus	Thermo Fisher Scientific	Cat# 12538120
NEBNext^®^ Poly(A) mRNA Magnetic Isolation Module	NEB	Cat# E7490S
NEBNext^®^ rRNA Depletion Kit	NEB	Cat# E7405
NEBNext^®^ Ultra II Directional RNA Library Prep Kit for Illumina	NEB	Cat# E7760S
NE-PER^™^ Nuclear and Cytoplasmic Extraction Reagents	Thermo Fisher Scientific	Cat# 78833
Q5 Site-Directed Mutagenesis Kit	NEB	Cat# E0554S
Venor GeM Mycoplasma Detection Kit, PCR-based	Sigma Aldrich	Cat# MP0025

Deposited data		

Zebrafish pA-selected RNA-seq	This paper	GEO: GSE271490
Zebrafish ribo-minus RNA-seq	This paper	GEO: GSE271491
Allelic series human melanoma cell lines pA-selected RNA-seq	This paper	GEO: GSE271489
pLENTI human melanoma cell lines pA-selected RNA-seq	This paper	GEO: GSE314384
pLENTI human melanoma cell lines ribo-minus RNA-seq	This paper	GEO: GSE314385
pLENTI human colon cancer cell lines pA-selected RNA-seq	This paper	GEO: GSE314387
PAR-CLIP	This paper	GEO: GSE271488

Experimental models: cell lines		

A375 human melanoma cells	ATCC	CRL-1619
SW480 colon adenocarcinoma cells	ATCC	CCL-228

Experimental models: organisms/strains		

Zebrafish: wild-type AB		NA
Zebrafish: Triple-mutant melanoma model (*p53^−/−^; mitfa:BRAF^V600E^; mitfa^−/−^)*	Ceol et al.^27^	NA
Zebrafish: Casper (*mitfa^−/−^; roy^−/−^*)	White et al.^68^	NA

Oligonucleotides		

gRNAs for ZC3H18 CRISPR, see Table S4	This paper	NA
Primers for qPCR, see Table S4	This paper	NA
gRNAs used for human and zebrafish CRISPRi, see Table S4	This paper	NA

Recombinant DNA		

Gecko2.0 vectors	Addgene, our lab	Plasmid #49535 (no longer available from Addgene)
pDestTol2CG2	Addgene	Plasmid #63156
MCR-eGFP	Addgene	Plasmid #118850
MCR-dCas9-KRAB-MeCP2	This paper, Addgene	Plasmid #251742
ZC3H18 coding sequence	Dharmacon	MHS6278-202759301, Clone ID: 6042036
pcDNA3.2 C-terminal V5 tag destination vectors	Thermo Fisher Scientific	Cat# 12489019
CRISPRi pXPR_066 all-in-one plasmid	Genetic Perturbation Platform (GPP), Broad Institute, Moore et al.^69^	NA
MCR-human HERVK3-int (exon-exon)	This paper	NA
MCR-human HERVK3-int (exon-intron)	This paper	NA
MCR-human MER4-int (exon-exon)	This paper	NA
MCR-human MER4-int (exon-intron)	This paper	NA
MCR-ZC3H18^trunc^	This paper	NA
MCR-zebrafish chr4:37858940 *BHIKHARI-2*	This paper	NA
MCR-zebrafish chr4:42592945 *BHIKHARI-2*	This paper	NA

Software and algorithms		

bedGraphToBigWig	Kent et al.^70^	NA
Bedtools (v. 2.30.0)	Quinlan et al.^71^	NA
Bowtie (v. 1.1.2)	Langmead et al.^72^	NA
CHOPCHOP	Labun et al.^73^	NA
CRISPOR	Concordet et al.^74^	NA
Cutadapt (v. 4.6)	NA	NA
DESeq2	Love et al.^33^	NA
DEXSeq	Anders et al.^75^	NA
featureCounts (v. 2.0.3)	Liao et al.^76^	NA
Fiji ImageJ	NA	NA
GraphPad Prism	NA	NA
Integrative Genomics Viewer (IGV)	Thorvaldsdottir et al.,^77^ Robinson et al.^29^	NA
liftOver	Hinrichs et al.^78^	NA
MACS2	NA	NA
MUSCLE	Edgar et al.^43^	NA
RepeatMasker GTF	UCSC	NA
SAMtools	Li et al.^79^	NA
Simple enrichment analysis (SEA)	Bailey et al.^49^	NA
SQuIRE	Yang et al.^28^	NA
STAR (v.2.5.3a)	Dobin et al.^80^	NA
StringTie (v.1.3.3b)	Pertea et al.^81^	NA
SWISS-MODEL server	Waterhouse et al.^82^	NA
wavClusteR (v. 2.36.0)	Comoglio et al.^48^	NA

Other		

Covaris E220 ultrasonicator	Covaris	NA
Nikon ECLIPSE TS2 inverted microscope	Nikon	NA
Nikon SMZ18 stereo microscope	Nikon	NA
Spectrolinker XL-1500	Spectro-UV	NA
Zeiss LSM 980 confocal microscope	ZEISS	NA

## Highlights

- Recurrent truncating mutations and loss of ZC3H18 occur in many human cancers
- ZC3H18 truncation promotes melanoma by stabilizing endogenous retroviral RNA
- ZC3H18-regulated retroviral RNA is sufficient to accelerate melanoma
- Endogenous retroviral RNA is required for ZC3H18^mut^-driven melanoma cell growth
