# Supplementary material for: Recurrent ZC3H18 mutations stabilize oncogenic endogenous retroviral RNA
Source: Cell Rep. Author manuscript; Available in PMC 2026 Aug 15. (PMC13476778; doi:10.1016/j.celrep.2026.117526)
Supplement: Supplementary file 1 [file NIHMS2199921-supplement-1.pdf]

Cell Reports, Volume *45*

Supplemental information

Recurrent ZC3H18 mutations stabilize oncogenic endogenous retroviral RNA

Tanzina Tanu, Anna M. Cox, Jennifer A. Karlow, Priyanka Sharma, Kaiwen Sun, Xueyang He, Swathy Babu, Madeline K. Roelofs, Ethan Jeon, Jiajia Chen, Constance Wu, Jared Brown, Nelson B. Barrientos, Andrew S. McCallion, Kevin M. Brown, Stephen J. Chanock, David Liu, Tongwu Zhang, Kathleen H. Burns, Paul L. Boutz, and Megan L. Insco

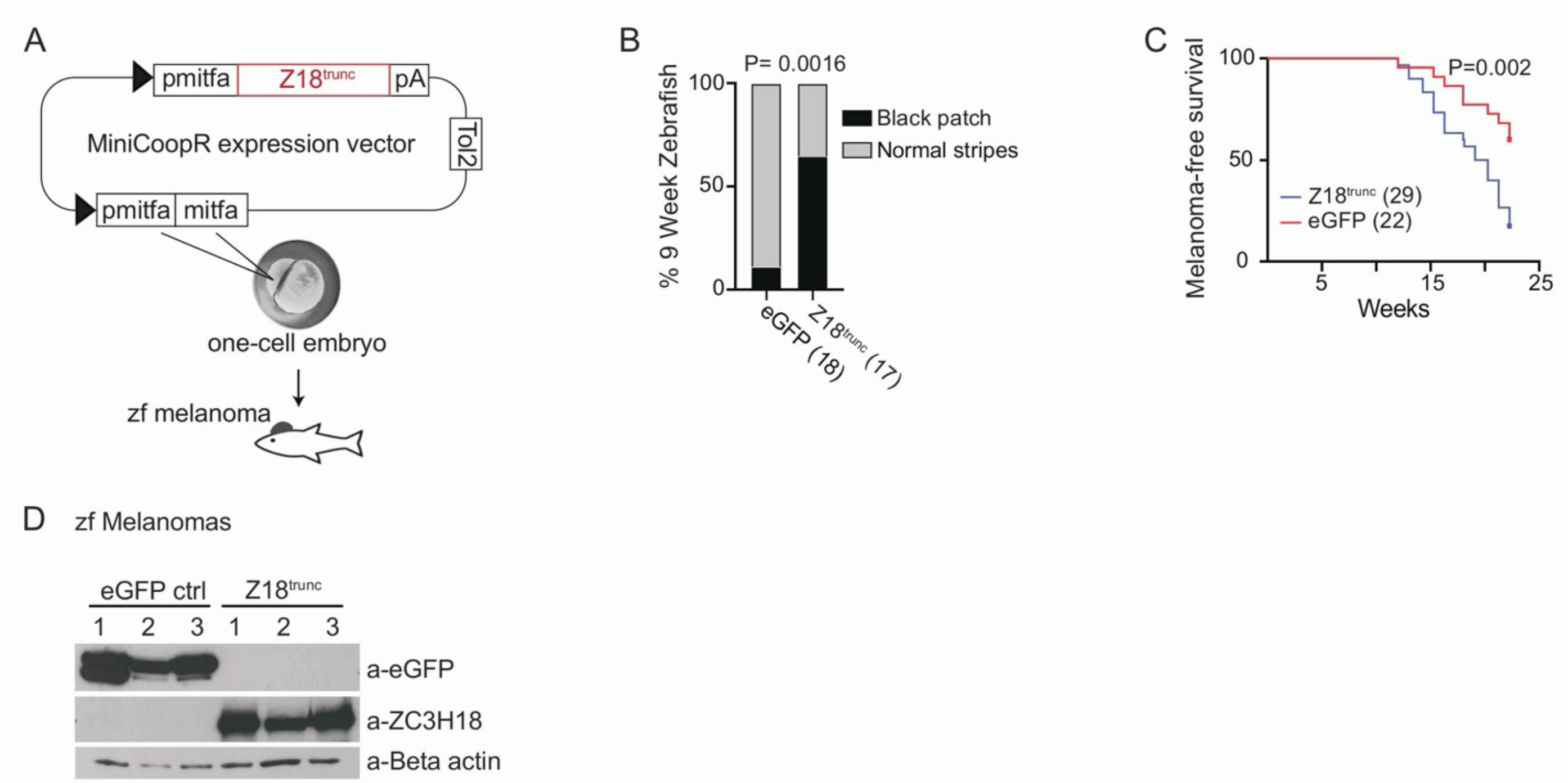

Fig. S1. ZC3H18^{trunc} Expression in Zebrafish Melanoma. Related to Fig. 1. A) Schematic of Z18^{trunc} expression in Triples using the MAZERATI system. pmitfa = melanocyte-specific promoter; Tol2 = sequences required in cis for transgenesis. B) Quantification of black patches. P=0.0016 (Fisher's exact test). n=zebrafish. C) Melanoma-free survival. P=0.002 (log-rank). n=zebrafish. D) Immunoblot of eGFP and human Z18^{trunc} in eGFP- and Z18^{trunc}-expressing zebrafish melanomas.

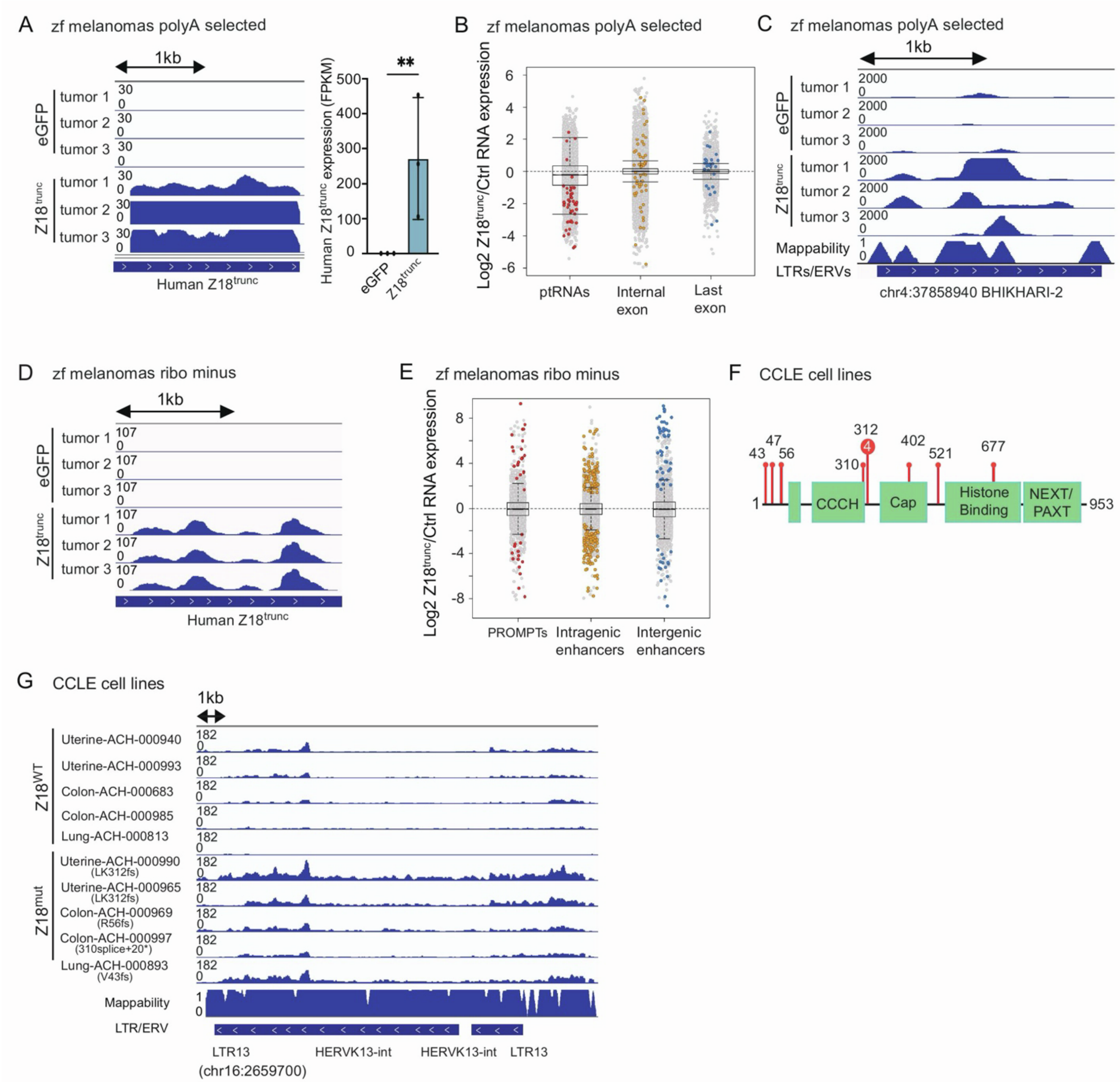

Fig. S2. RNA-seq Defines ZC3H18^{trunc} RNA Targets. Related to Fig. 2. A) IGV plot (left) and FPKM quantification (right) of Z18^{trunc} expression from pA-selected RNA-seq of eGFP- and Z18^{trunc}-expressing zebrafish melanomas. Mean ± SD. **P=0.0018. (log-normal Welch's t-test). B) Box plots of log2 fold change (Z18^{trunc} versus eGFP zebrafish melanomas) for ptRNAs (left), internal exons (middle), or last exons (right). Colored dots indicate q<0.1. ptRNA = RNA generated by usage of intronic polyadenylation sites. Internal exon = constitutive internal exon. C) IGV plot of a representative *bhik-2* ERV locus in eGFP- and Z18^{trunc}-expressing zebrafish melanomas. D) IGV plot of Z18^{trunc} expression from ribo-minus RNA-seq in eGFP- and Z18^{trunc}-expressing zebrafish melanomas. E) Box plots of log2 fold change (Z18^{trunc} versus eGFP) for promoter upstream transcripts (PROMPTs) (left), intragenic enhancers (middle), or intergenic enhancers (right). Colored dots indicate q<0.1. F) Lollipop plot of Z18 mutations in CCLE lines (n=11). G) IGV plots of LTR/ERV expression in Z18^{mut} and matched Z18^{WT} CCLE lines. Box plot conventions: median (black line), interquartile range (IQR) (box), 1.5xIQR (whiskers).

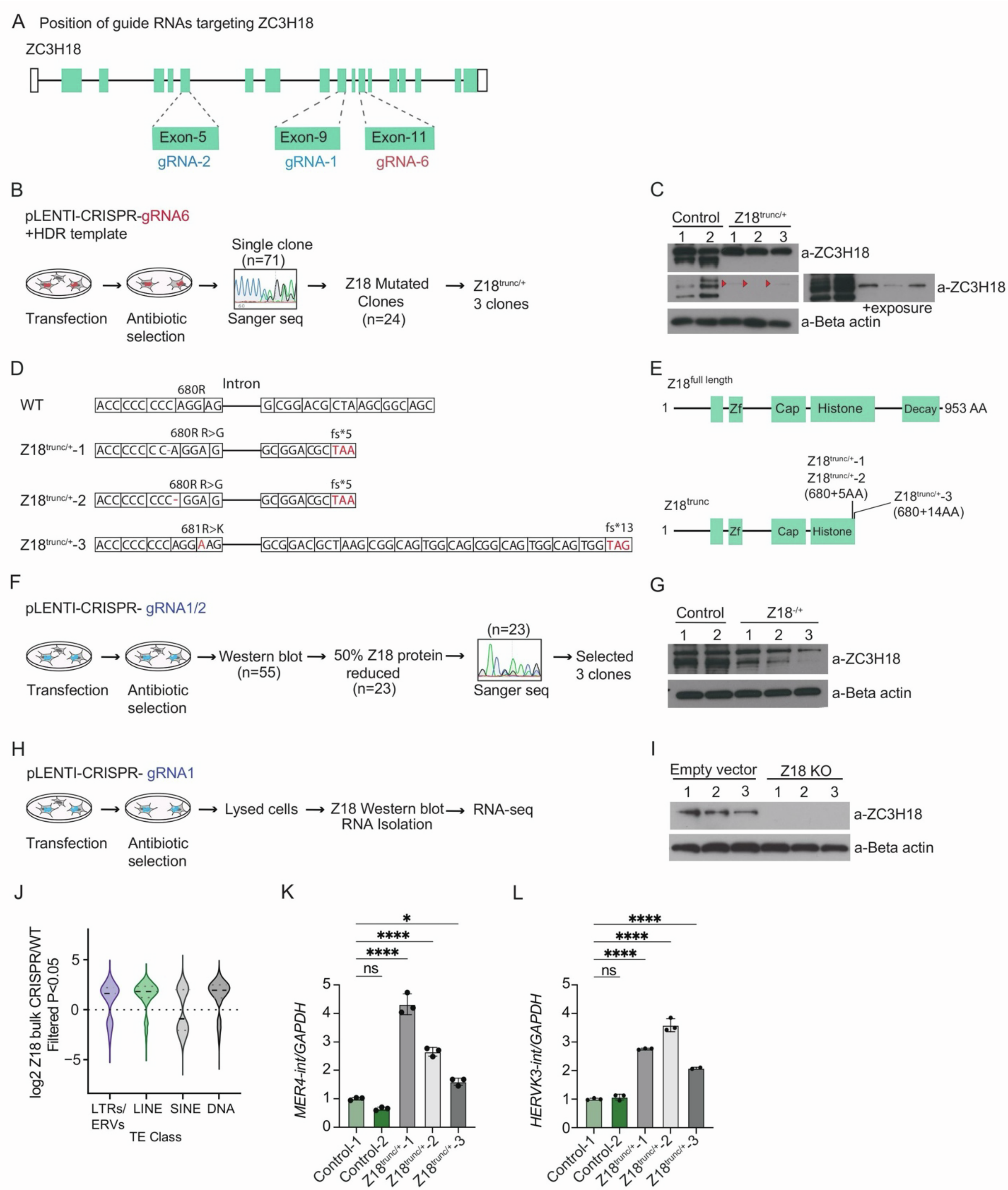

A Position of guide RNAs targeting ZC3H18
ZC3H18
Exon-5
gRNA-2
Exon-9
gRNA-1
Exon-11
gRNA-6
B
pLENTI-CRISPR-gRNA6
+HDR template
Single clone
(n=71)
Transfection
Antibiotic selection
Sanger seq
Z18 Mutated Clones
(n=24)
Z18trunc/+
3 clones
C
Control
Z18trunc/+
a-ZC3H18
+exposure
a-Beta actin
D
WT
680R
Intron
680R R>G
fs*5
681R>K
fs*13
Z18trunc/+-1
Z18trunc/+-2
Z18trunc/+-3
E
Z18full length
Zf
Cap
Histone
Decay
953 AA
Z18trunc
(680+5AA)
(680+14AA)
F
pLENTI-CRISPR- gRNA1/2
Western blot
(n=55)
50% Z18 protein reduced
(n=23)
Sanger seq
Selected 3 clones
G
Control
Z18-/+
H
pLENTI-CRISPR- gRNA1
Lysed cells
Z18 Western blot
RNA Isolation
RNA-seq
I
Empty vector
Z18 KO
J
log2 Z18 bulk CRISPR/WT Filtered P<0.05
LTRs/ ERVs
LINE
SINE
DNA
TE Class
K
MER4-int/GAPDH
Control-1
Control-2
L
HERVK3-int/GAPDH
ns
****
*

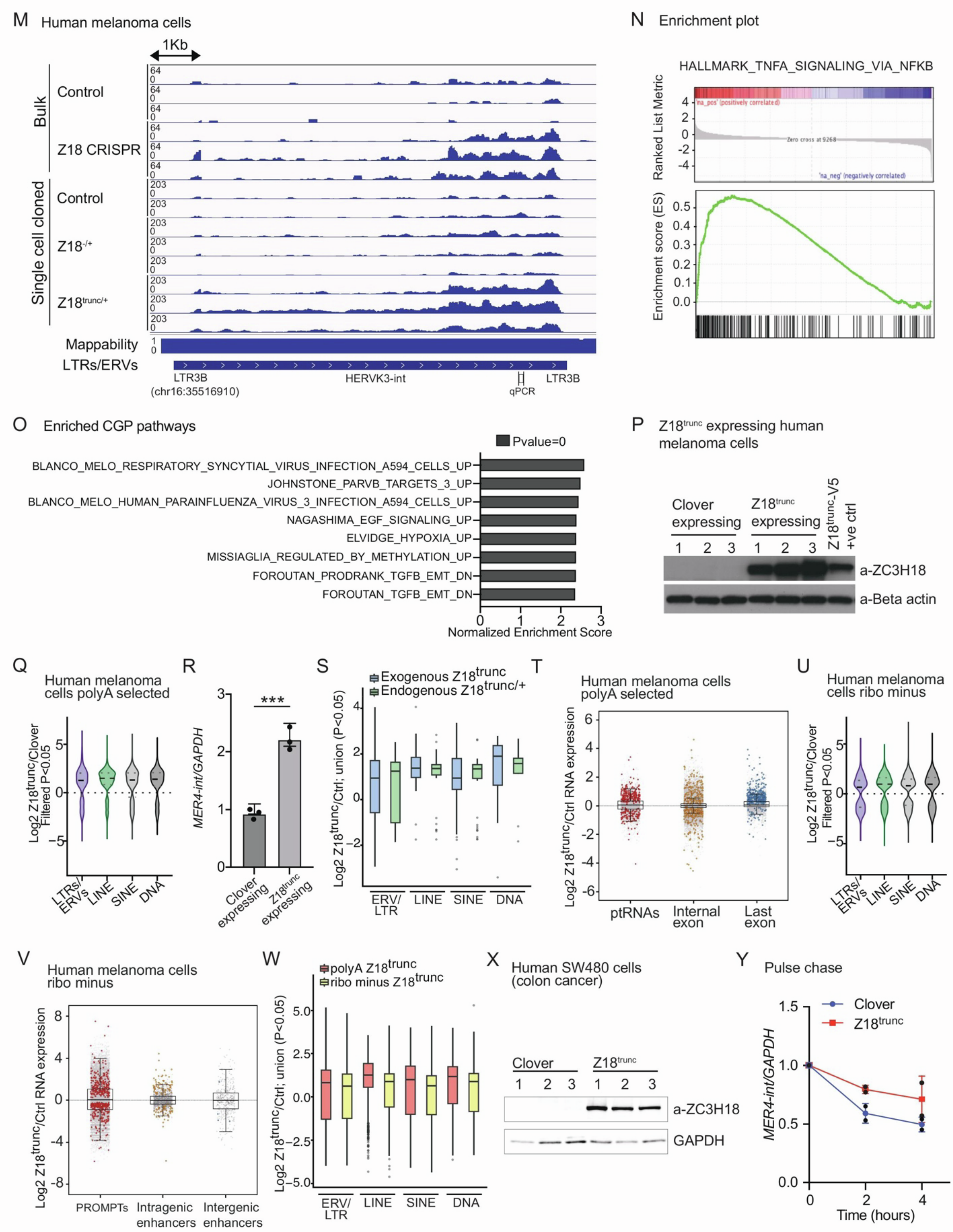

M Human melanoma cells
1Kb
Bulk
Control
Z18 CRISPR
Single cell cloned
Control
Z18-/+
Z18trunc/+
Mappability
LTRs/ERVs
LTR3B
(chr16:35516910)
HERVK3-int
qPCR
LTR3B
N Enrichment plot
HALLMARK_TNFA_SIGNALING_VIA_NFKB
Ranked List Metric
Enrichment score (ES)
O Enriched CGP pathways
Pvalue=0
BLANCO_MELO_RESPIRATORY_SYNCYTIAL_VIRUS_INFECTION_A594_CELLS_UP
JOHNSTONE_PARVB_TARGETS_3_UP
BLANCO_MELO_HUMAN_PARAINFLUENZA_VIRUS_3_INFECTION_A594_CELLS_UP
NAGASHIMA_EGF_SIGNALING_UP
ELVIDGE_HYPOXIA_UP
MISSIAGLIA_REGULATED_BY_METHYLATION_UP
FOROUTAN_PRODRANK_TGFB_EMT_DN
FOROUTAN_TGFB_EMT_DN
Normalized Enrichment Score
P Z18trunc expressing human melanoma cells
Clover expressing
Z18trunc expressing
Z18trunc_V5 +ve ctrl
a-ZC3H18
a-Beta actin
Q Human melanoma cells polyA selected
Log2 Z18trunc/Clover Filtered P<0.05
LTRs/ERVs
LINE
SINE
DNA
R
MER4-int/GAPDH
Clover expressing
Z18trunc expressing
S
Exogenous Z18trunc
Endogenous Z18trunc/+
Log2 Z18trunc/Ctrl; union (P<0.05)
ERV/LTR
LINE
SINE
DNA
T Human melanoma cells polyA selected
Log2 Z18trunc/Ctrl RNA expression
ptRNAs
Internal exon
Last exon
U Human melanoma cells ribo minus
Log2 Z18trunc/Clover Filtered P<0.05
V Human melanoma cells ribo minus
Log2 Z18trunc/Ctrl RNA expression
PROMPTs
Intragenic enhancers
Intergenic enhancers
W
polyA Z18trunc
ribo minus Z18trunc
Log2 Z18trunc/Ctrl; union (P<0.05)
X Human SW480 cells (colon cancer)
Clover
Z18trunc
a-ZC3H18
GAPDH
Y Pulse chase
Clover
Z18trunc
MER4-int/GAPDH
Time (hours)

Fig. S3. Validation of ZC3H18 Mutations in Human Melanoma Cell Lines. Related to Fig. 3. A) Schematic of sgRNA target positions in *Z18*. B) Workflow for creating Z18$^{trunc/+}$ cell lines. C) Z18 immunoblot of Z18^{WT} control and Z18$^{trunc/+}$ cell lines. Red arrows = truncated isoform; longer exposure on right. D-E) Diagram showing the effects of Z18^{trunc} mutations on D) RNA and E) protein. F-G) Z18^{WT} control and heterozygous loss-of-function Z18$^{-/+}$ cell line F) workflow and G) immunoblot. H-I) bulk Z18 CRISPR versus control H) workflow and I) immunoblot. J) Log2 fold change of significantly changed TEs (P<0.05) from bulk Z18 CRISPR (n=3) versus control (n=3). K-L) qPCR for two LTRs/ERVs versus GAPDH from control (n=2) and Z18$^{trunc/+}$ (n=3) cell lines. Mean ± SD. *P=0.01, ****P<0.0001, ns=non-significant, (one-way ANOVA). M) IGV plot of chr16 *HERVK3-int* expression across the allelic series. N-O) GSEA of Z18$^{trunc/+}$ (n=3) versus Z18^{WT} (n=2) pA-selected RNA-seq: N) top Hallmark pathway enrichment plot; O) normalized enrichment scores for top CGP pathways. P) Z18 immunoblot from Clover- and Z18^{trunc}-expressing human melanoma cell lines. Q) Log2 fold change of significantly changed TE expression (P<0.05) in Z18^{trunc}-expressing (n=3) versus control (n=3) melanoma lines from pA-selected RNA-seq. R) qPCR of *MER4-int* (primer location, Fig. 3F) in Clover- (n=3) and Z18^{trunc}-expressing (n=3) lines. Mean ± SD. ***P=0.0005 (two-sided unpaired t-test). S) Log2 fold change Z18^{trunc}/control for the union of significantly altered transposable elements (P < 0.05), grouped by TE class, in exogenous Z18^{trunc}-expressing cell lines (blue) and endogenous Z18$^{trunc/+}$ cell lines (green). T) Log2 fold change Z18^{trunc}/Clover for ptRNAs (left), internal exons (middle), or last exons (right). Colored dots indicate q<0.1. ptRNA = RNA generated by usage of intronic polyadenylation sites. Internal exon = constitutive internal exon. U) Log2 fold change Z18^{trunc}-expressing (n=3) versus control (n=3) A375 human melanoma cell lines for significantly changed (P<0.05) TEs from ribo-minus RNA-seq. V) Log2 fold change Z18^{trunc}/Clover for promoter upstream transcripts (PROMPTs) (left), intragenic enhancers (middle), or intergenic enhancers (right) measured from ribo-minus RNA-seq. Colored dots indicate q<0.1. W) Log2 fold change Z18^{trunc}/Clover comparing polyA (red) versus ribo-minus (yellow) RNA-seq for the union of significantly altered (P < 0.05) TEs by class. X) Z18 immunoblot of Clover- and Z18^{trunc}-expressing human SW480 colon cancer cells. Y) *MER4-int* decay by 4sU pulse-chase qPCR for Z18^{trunc}- (n=2) versus Clover-expressing (n=2) melanoma lines (primer location, Fig. 3F). Mean ± SD. For line 1, Z18^{trunc}- versus Clover-expressing: two hours, **P=0.009; four hours, non-significant. For line 2, Z18^{trunc}- versus Clover-expressing: two hours ***P=0.0006; four hours, non-significant (Welch's t-test). Box plot conventions: median (black line), IQR (box), whiskers (1.5x IQR). Violin plot conventions: Thick dotted line = median. Light dotted line = quartiles.

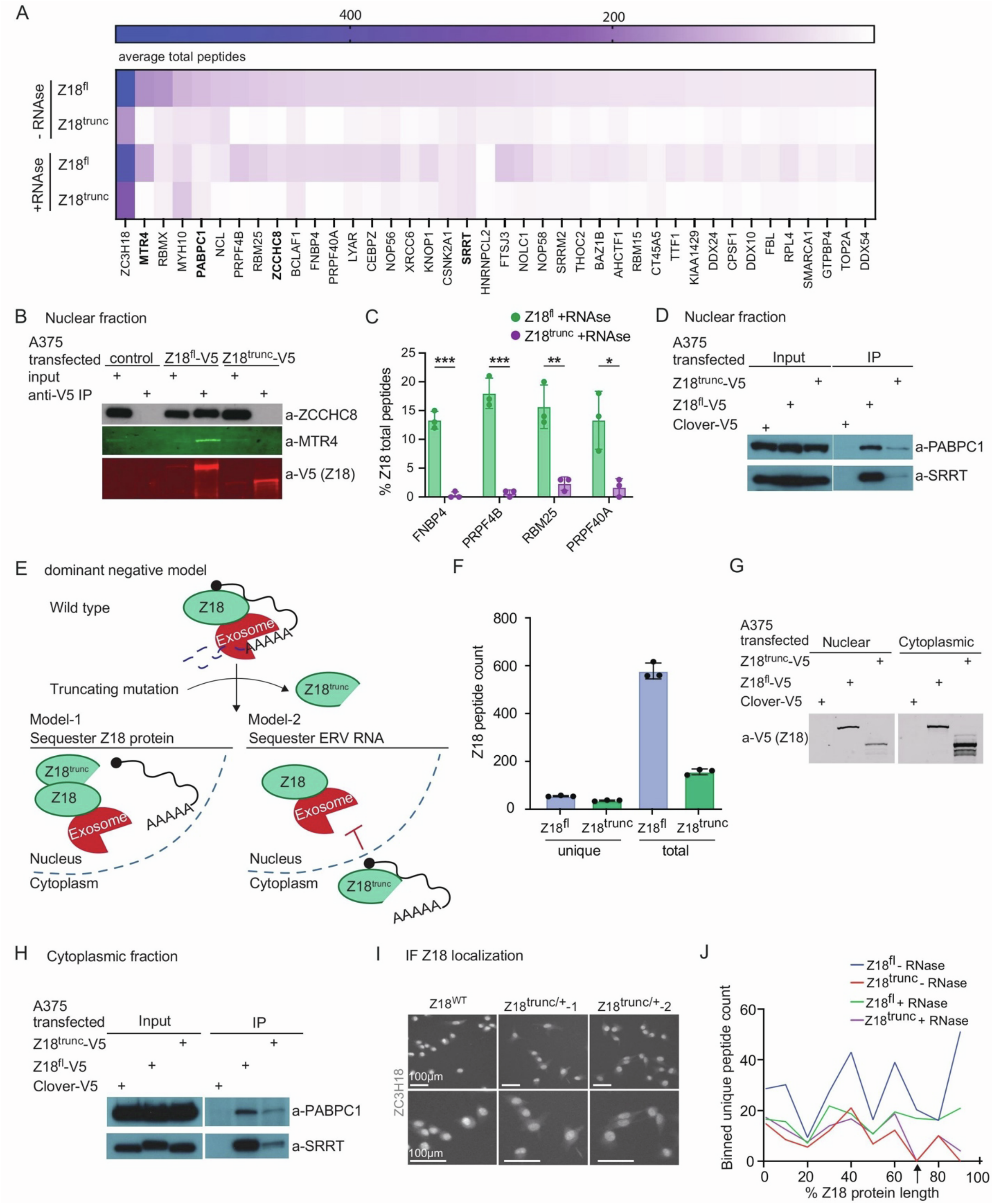
A
400
200
average total peptides
- RNAse
+RNAse
Z18fl
Z18trunc
ZC3H18
MTR4
RBMX
MYH10
PABPC1
NCL
PRPF4B
RBM25
ZCCHC8
BCLAF1
FNBP4
PRPF40A
LYAR
CEBPZ
NOP56
XRCC6
KNOP1
CSNK2A1
SRRT
HNRNPCL2
FTSJ3
NOLC1
NOP58
SRRM2
THOC2
BAZ1B
AHCTF1
RBM15
CT45A5
TTF1
KIAA1429
DDX24
CPSF1
DDX10
FBL
RPL4
SMARCA1
GTPBP4
TOP2A
DDX54
B Nuclear fraction
A375
transfected
control
Z18fl-V5
Z18trunc-V5
input
anti-V5 IP
a-ZCCHC8
a-MTR4
a-V5 (Z18)
C
Z18fl +RNAse
Z18trunc +RNAse
% Z18 total peptides
***
***
**
*
FNBP4
PRPF4B
RBM25
PRPF40A
D Nuclear fraction
A375
transfected
Input
IP
Z18trunc-V5
Z18fl-V5
Clover-V5
a-PABPC1
a-SRRT
E dominant negative model
Wild type
Z18
Exosome
AAAAA
Truncating mutation
Z18trunc
Model-1
Sequester Z18 protein
Model-2
Sequester ERV RNA
Nucleus
Cytoplasm
F
Z18 peptide count
Z18fl
Z18trunc
unique
total
G
A375
transfected
Nuclear
Cytoplasmic
Z18trunc-V5
Z18fl-V5
Clover-V5
a-V5 (Z18)
H Cytoplasmic fraction
A375
transfected
Input
IP
Z18trunc-V5
Z18fl-V5
Clover-V5
a-PABPC1
a-SRRT
I IF Z18 localization
Z18WT
Z18trunc/+-1
Z18trunc/+-2
ZC3H18
100μm
J
Z18fl - RNase
Z18trunc - RNase
Z18fl + RNase
Z18trunc + RNase
Binned unique peptide count
% Z18 protein length

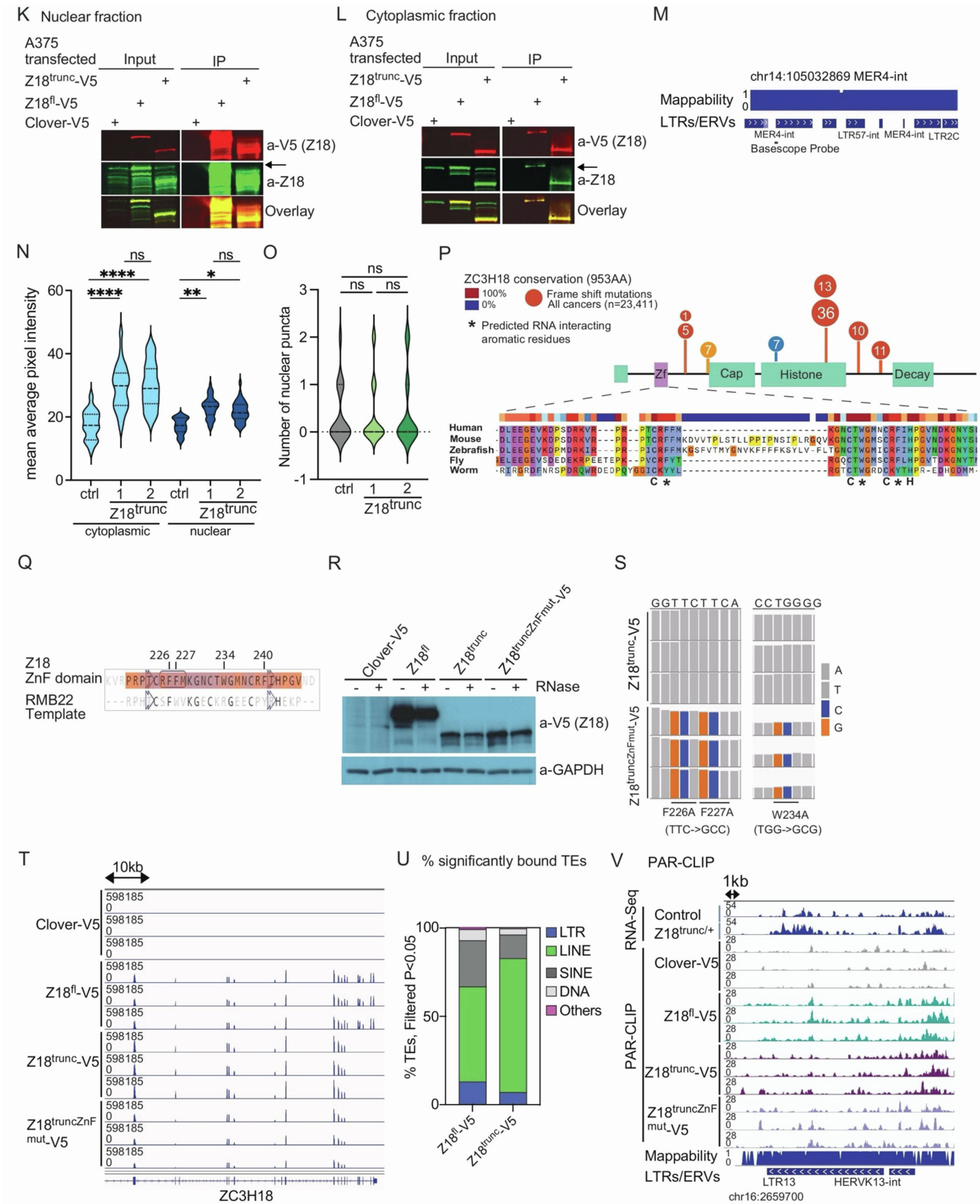

K Nuclear fraction
A375 transfected
Input
IP
Z18trunc-V5
Z18fl-V5
Clover-V5
a-V5 (Z18)
a-Z18
Overlay
L Cytoplasmic fraction
M
chr14:105032869 MER4-int
Mappability
LTRs/ERVs
MER4-int
LTR57-int
LTR2C
Basescope Probe
N
mean average pixel intensity
ns
****
*
**
ctrl
Z18trunc
cytoplasmic
nuclear
O
Number of nuclear puncta
P
ZC3H18 conservation (953AA)
100%
0%
Frame shift mutations All cancers (n=23,411)
* Predicted RNA interacting aromatic residues
Zf
Cap
Histone
Decay
Human
Mouse
Zebrafish
Fly
Worm
Q
Z18 ZnF domain
RMB22 Template
226
227
234
240
R
Clover-V5
Z18fl
Z18trunc
Z18truncZnFmut-V5
RNase
a-GAPDH
S
GGTTCTTCA
CCTGGGG
Z18truncZnFmut-V5
F226A F227A (TTC->GCC)
W234A (TGG->GCG)
T
10kb
598185
Z18truncZnF mut-V5
ZC3H18
U % significantly bound TEs
% TEs, Filtered P<0.05
LTR
LINE
SINE
DNA
Others
V PAR-CLIP
1kb
RNA-Seq
Control
Z18trunc/+
PAR-CLIP
LTR13
HERVK13-int
chr16:2659700

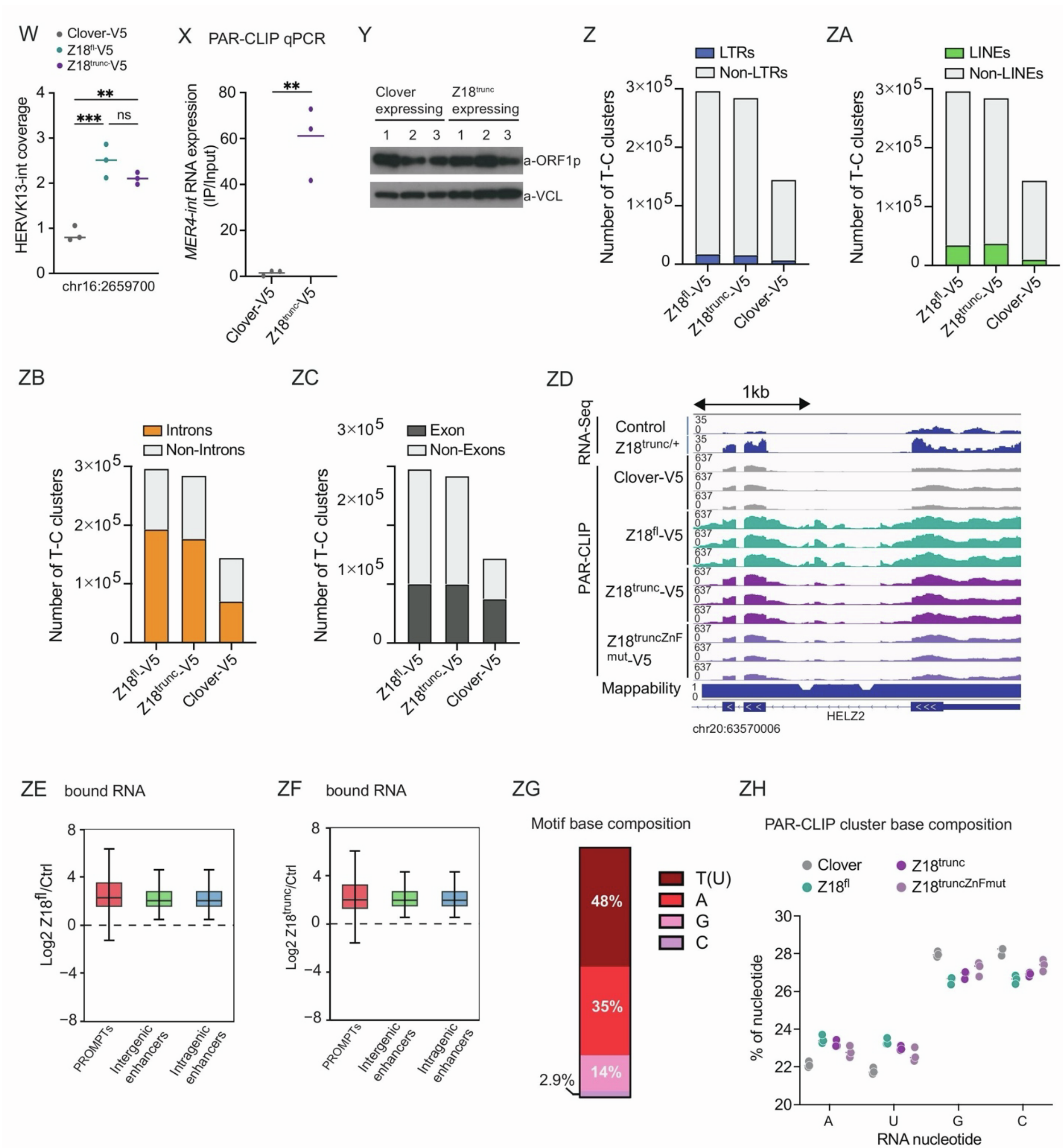

Fig. S4. Dominant-Negative Model for ZC3H18^{trunc}. Related to Fig. 4. A) Heat map of average total peptides from immunoprecipitation mass spectrometry (IP-MS) of V5-tagged Z18^{fl} and Z18^{trunc} ± RNase. n=3, all conditions. Top 40 of 240 proteins displayed. B) V5 IP from nuclear extracts; immunoblot for ZCCHC8, MTR4, and V5 from cells expressing V5-tagged Clover, Z18^{fl}, or Z18^{trunc}. C) Total peptide counts normalized to Z18 for four additional significantly differentially bound proteins in Z18^{fl} versus Z18^{trunc}. Mean ± SD. FNBP4 ***P=0.0002, PRPF4B ***P=0.00034, RBM25 **P=0.0032, PRPF40A *P=0.015, (two-sided t-test). n=3, all conditions. D) V5 IP from nuclear extracts; immunoblot detecting PABPC1 and SRRT from cells expressing V5-tagged Clover, Z18^{fl}, or Z18^{trunc}. E) Schematic depicting two models of Z18^{trunc} dominant negative activity. F) IP-MS unique and total Z18 peptide counts. Mean ± SD. n=3, all conditions. G) V5 IP and immunoblot from

nuclear or cytoplasmic fractions of cells expressing V5-tagged Clover, Z18^{fl}, or Z18^{trunc}. H) V5 IP from cytoplasmic extracts; immunoblot detecting PABPC1 and SRRT from cells expressing V5-tagged Clover, Z18^{fl}, or Z18^{trunc}. I) IF of ZC3H18 in Z18^{WT}, Z18$^{trunc/+}$ line1, and Z18$^{trunc/+}$ line2. Representative images. Scale bar = 100μm. J) IP-MS binned average total Z18 peptide counts by % Z18 protein length. n=3 IPs, all conditions. K-L) V5 IP and immunoblot for V5 and Z18 from cells expressing V5-tagged Clover, Z18^{fl}, or Z18^{trunc} from K) nuclear or L) cytoplasmic extracts. Arrows indicate that Z18^{trunc} does not co-IP endogenous Z18^{fl}. M) BaseScope *MER4-int* probe location. N) Average *MER4-int* pixel intensity in A375s (n=18 cells), Z18^{trunc} line1 (n=48 cells), and line2 (n=35 cells). *P=0.0302, **P=0.0013, ****P<0.0001, ns=non-significant (one-way ANOVA). O) Number of nuclear puncta per cell in A375s (n=18), Z18^{trunc} line1 (n=48), Z18^{trunc} line2 (n=35). ns=non-significant (Ordinary one-way ANOVA). P) Protein alignment of the Z18 zinc-finger across species. C-C-C-H = residues predicted to coordinate with zinc to form the zinc-finger domain structure. *=aromatic residues predicted to be involved in RNA coordination. Q) Target-template protein sequence alignment for Z18 homology modeling. R) Immunoblot for V5 or GAPDH from PAR-CLIP input lysates from cells expressing V5-tagged Clover, Z18^{fl}, Z18^{trunc}, or Z18^{trunc} ZnF aromatic mutant (Z18^{truncZnFmut}) after 4sU labeling and crosslinking. S-T) IGV plots from PAR-CLIP RNA-seq: S) Z18^{truncZnFmut} (F226A, F227A, and W234A) mutations versus Z18^{trunc}; T) transgene expression verifying length of Z18^{fl}, Z18^{trunc}, or Z18^{truncZnFmut}. n=3 replicates, all conditions. U) Percentage of significantly bound (P<0.05) TEs for Z18^{fl}-V5 or Z18^{trunc}-V5 versus Clover from PAR-CLIP SQuIRE analysis. V-W) PAR-CLIP *HERVK13-int.* V) IGV visualization and W) coverage quantification from Z18^{fl}, Z18^{trunc}, and control Clover. ***P=0.0005, **P=0.0021, ns=non-significant (one-way ANOVA). X) V5 PAR-CLIP qPCR of *MER4-int* RNA from Clover-V5 or Z18^{trunc}-V5 expressing cells. **P=0.0032, (unpaired two-sided t-test). Y) LINE1 ORF1p immunoblot from Clover- and Z18^{trunc}-expressing human melanoma cell lines. n=independently-derived lines. Z-ZC) Merged clusters of T-to-C transitions (wavClusteR) from Z18^{fl}-V5, Z18^{trunc}-V5, and Clover-V5 annotated for overlap with Z) LTR/ERVs, ZA) LINEs, ZB) introns, and ZC) exons. Clover clusters represent non-specific RNA binding (background). ZD) IGV plot of Z18-bound intron-containing transcript. ZE-ZF) PAR-CLIP log2 fold-enrichment (Z18 versus Clover) for promoter upstream transcripts (PROMPTs) (left), intragenic enhancers (middle), or intergenic enhancers (right) for ZE) Z18^{fl} and ZF) Z18^{trunc}. Mean ± SD. In box plots, the black horizontal line indicates the median, the box covers the interquartile range (IQR), and the whiskers extend to 1.5× the IQR. ZG) Nucleotide quantification from top SEA-enriched Z18 motifs from PAR-CLIP (Table S6). ZH) Nucleotide quantification in T-to-C transition clusters from PAR-CLIP. Clover represents base composition of non-specific RNA binding (background). n=3 replicates, all conditions.

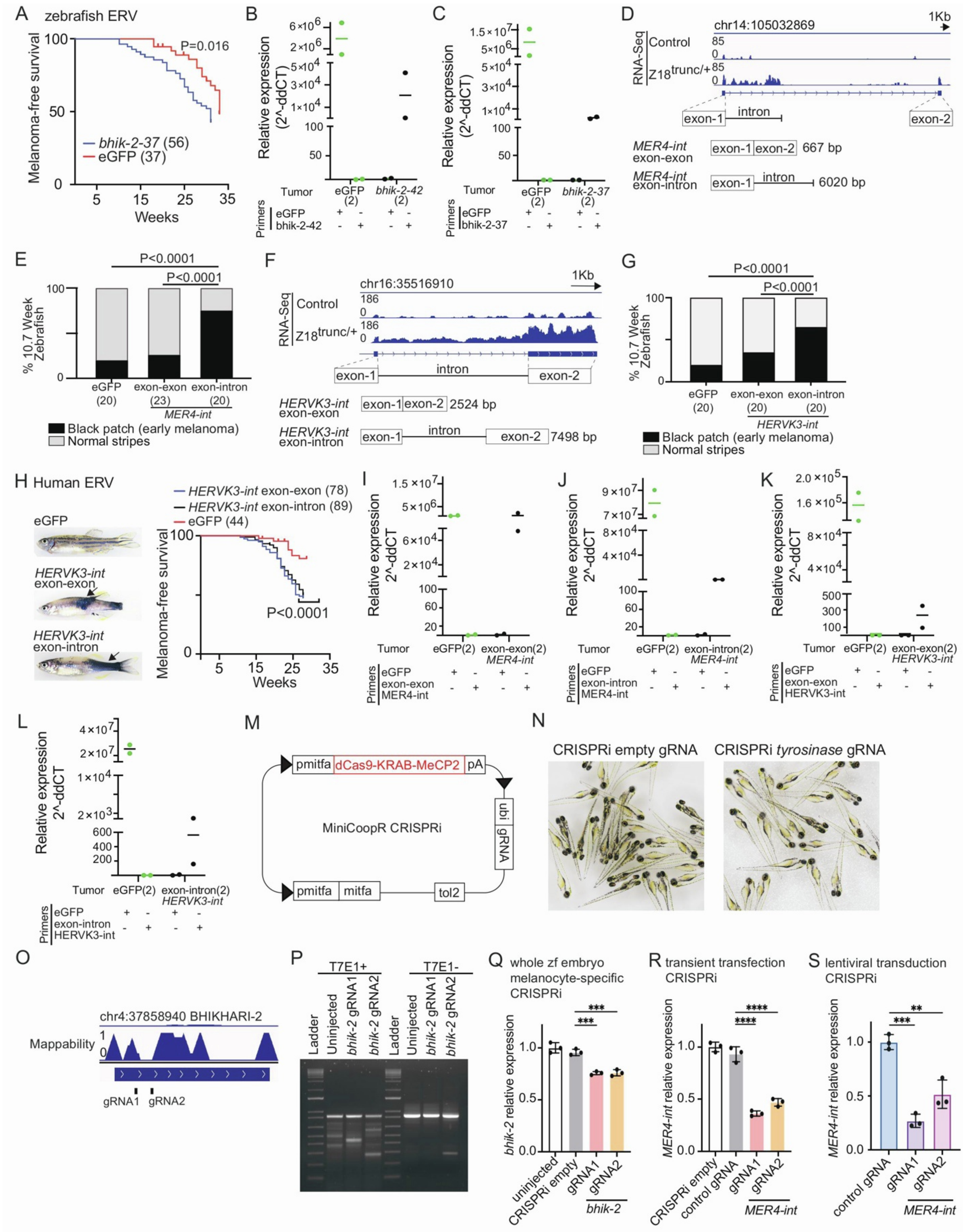
A zebrafish ERV
Melanoma-free survival
P=0.016
bhik-2-37 (56)
eGFP (37)
Weeks
B
Relative expression (2^-ddCT)
Tumor eGFP (2) bhik-2-42 (2)
Primers eGFP bhik-2-42
C
Relative expression (2^-ddCT)
Tumor eGFP (2) bhik-2-37 (2)
Primers eGFP bhik-2-37
D
chr14:105032869
1Kb
RNA-Seq Control
Z18trunc/+
exon-1 intron exon-2
MER4-int exon-exon exon-1 exon-2 667 bp
MER4-int exon-intron exon-1 intron 6020 bp
E
% 10.7 Week Zebrafish
P<0.0001
P<0.0001
eGFP (20) exon-exon (23) exon-intron (20)
MER4-int
Black patch (early melanoma)
Normal stripes
F
chr16:35516910
1Kb
RNA-Seq Control
Z18trunc/+
exon-1 intron exon-2
HERVK3-int exon-exon exon-1 exon-2 2524 bp
HERVK3-int exon-intron exon-1 intron exon-2 7498 bp
G
% 10.7 Week Zebrafish
P<0.0001
P<0.0001
eGFP (20) exon-exon (20) exon-intron (20)
HERVK3-int
Black patch (early melanoma)
Normal stripes
H Human ERV
eGFP
HERVK3-int exon-exon
HERVK3-int exon-intron
HERVK3-int exon-exon (78)
HERVK3-int exon-intron (89)
eGFP (44)
Melanoma-free survival
P<0.0001
Weeks
I
Relative expression 2^-ddCT
Tumor eGFP(2) exon-exon(2) MER4-int
Primers eGFP exon-exon MER4-int
J
Relative expression 2^-ddCT
Tumor eGFP(2) exon-intron(2) MER4-int
Primers eGFP exon-intron MER4-int
K
Relative expression 2^-ddCT
Tumor eGFP(2) exon-exon(2) HERVK3-int
Primers eGFP exon-exon HERVK3-int
L
Relative expression 2^-ddCT
Tumor eGFP(2) exon-intron(2) HERVK3-int
Primers eGFP exon-intron HERVK3-int
M
pmitfa dCas9-KRAB-MeCP2 pA
ubi gRNA
MiniCoopR CRISPRi
pmitfa mitfa
tol2
N
CRISPRi empty gRNA
CRISPRi tyrosinase gRNA
O
chr4:37858940 BHIKHARI-2
Mappability
gRNA1 gRNA2
P
T7E1+ T7E1-
Ladder Uninjected bhik-2 gRNA1 bhik-2 gRNA2 Ladder Uninjected bhik-2 gRNA1 bhik-2 gRNA2
Q whole zf embryo melanocyte-specific CRISPRi
bhik-2 relative expression
uninjected CRISPRi empty gRNA1 gRNA2
bhik-2
R transient transfection CRISPRi
MER4-int relative expression
CRISPRi empty control gRNA gRNA1 gRNA2
MER4-int
S lentiviral transduction CRISPRi
MER4-int relative expression
control gRNA gRNA1 gRNA2
MER4-int

Fig. S5. CRISPR Inhibition Decreases Z18-regulated ERV RNA in Zebrafish and Human Cells. Related to Fig. 5. A) Percent melanoma-free survival for Triples zebrafish with melanocyte-specific expression of eGFP or *bhik-2-37*. P=0.016 (log-rank). n=zebrafish. B-C) qPCR of eGFP and *bhik-2* (normalized to *gapdh)* from eGFP- and *bhik-2*-expressing zebrafish melanomas for B) *bhik-2-42* and C) *bhik-2-37.* n=melanomas. D) Schematic showing *MER4-int* exon-exon and exon-intron transcripts. E) Quantification of black patches. P<0.0001 (Fisher's exact test). n=zebrafish. F) Schematic showing *HERVK3-int* exon-exon and exon-intron transcripts. G) Quantification of black patches. P<0.0001 (Fisher's exact test). n=zebrafish. H) Photos or melanoma-free survival for Triples zebrafish with melanocyte-specific expression of eGFP or the *HERVK3-int* exon-exon and exon-intron transcripts. P<0.0001 (log-rank). n=zebrafish. I-L) qPCR of eGFP and ERVs (normalized to *gapdh)* from eGFP or human ERV-expressing zebrafish melanomas: I) *MER4-int* exon-exon, J) *MER4-int* exon-intron, K) *HERVK3-int* exon-exon, L) *HERVK3* exon-intron. M) Schematic of the melanocyte-specific zebrafish codon-optimized MiniCoopR CRISPRi plasmid. pmitfa = melanocyte-specific promoter; Tol2 = sequences required in cis for transgenesis. N) Images of 6 dpf wild-type zebrafish embryos injected with the melanocyte-specific CRISPRi with or without a gRNA targeting *tyrosinase.* O) Schematic showing gRNA position targeting *bhik-2-37*. gRNA-1 and gRNA-2 target 13/15 top Z18-regulated *bhik-2* elements. P) T7E1 of *bhik-2-42* PCR from genomic DNA isolated from zebrafish embryos: 1) uninjected, 2) *bhik-2* gRNA1-injected, or 3) *bhik-2* gRNA2-injected. Q) qPCR for *bhik-2-42* relative to *gapdh* from Triples embryos: either uninjected or injected with melanocyte-specific CRISPRi: 1) empty, 2) *bhik-2* gRNA1, 3) *bhik-2* gRNA2. gRNA1 ** P=0.0002, gRNA2 ***P=0.0003 (one-way ANOVA). R-S) qPCR for *MER4-int* relative to *GAPDH* in A375 cells. R) transient CRISPRi: 1) empty, 2) control gRNA, 3) *MER4-int* gRNA1, or 4) *MER4-int* gRNA2. ****P<0.0001 (one-way ANOVA). S) lentiviral CRISPRi: 1) control gRNA, 2) *MER4-int* gRNA1, or 3) *MER4-int* gRNA2. gRNA1 ***P=0.0002, gRNA2 **P=0.0017 (one-way ANOVA).

Table S1. MSI status of $Z18^{mut}$ and $Z18^{WT}$ TCGA tumors.

MSI score	Z18 mutations	$Z18^{WT}$
MSI high (>10)	75	244
MSI not high (<10)	137	10326

Table S2. Average TE expression and statistics of TE classes from zebrafish pA-selected RNA-seq. TE classes are ranked by the average expression difference between $Z18^{trunc}$ and eGFP.

CLASS	MEAN EXPRESSION (eGFP)	MEAN EXPRESSION ($Z18^{trunc}$)	WILCOXON SIGNED-RANK TEST P-VALUE	WELCH'S T-TEST P-VALUE
LTR/ERV	0.3716	0.5546	4.54e-12	0.00269
SINE	0.3414	0.3890	1.34e-14	0.538
LINE	0.2124	0.2304	0.000841	0.501
DNA	0.4096	0.4055	<2.2e-16	0.814

Table S3. Medians for Fig. S2E from ribo-minus RNA-seq in $Z18^{trunc}$ versus eGFP zebrafish melanoma.

	**Medians**		
$Z18^{trunc}$ versus eGFP	PROMPTs	Intragenic enhancer	Intergenic enhancer
	-0.038	-0.023	-0.047

Table S4. Nucleic acid sequences.

gRNAs	Sequence 5′-3′ (underlined=NGG)	Strand	Z18 Exon	Species
Z18 gRNA1	ATGAAGACCGGGATCGCGACCGG	-	9	Human
Z18 gRNA2	TGACCATTGGCGAAGACGAACGG	+	5	Human
Z18 gRNA6	CGCCGGAGTGCTCACCTCCTGGG	-	11	Human
tyrosinase gRNA	AGATGCTTTACACACTGCTGG	-	na	Zebrafish
bhik-2 gRNA1	CATTGATTAACCTAGTTGATTGG	-	na	Zebrafish
bhik-2 gRNA2	CCCAAGGCGAGCACATGACCAGG	+	na	Zebrafish
control gRNA	AACAATAGGCACGTGACCGCAGG	Intergenic	na	Human
MER4-int gRNA1	GAATCAGGGCGAGTACATAAAGG	+	na	Human
MER4-int gRNA2	ATCTCGCACAAGAAAGAATCAGG	+	na	Human

qPCR Primers		5′-3′ sequence
qPCR-MER4-int	Fw	CTTGGGGTCTTGATCGGGAC
qPCR-MER4-int	Rev	ATCTGCCTTCCACCGTCATG
qPCR-chr16 HERVK3-int	Fw	TCAGCTTACTCATGGCACTCGT
qPCR-chr16 HERVK3-int	Rev	TGAGGACACCACAACAGAGGG
qPCR-GAPDH	Fw	CAAGAGCACAAGAGGAAGAGAG
qPCR-GAPDH	Rev	CTACATGGCAACTGTGAGGAG
qPCR- zebrafish gapdh	Fw	TTCACAGGTTTACATGTTCCAGT
qPCR- zebrafish gapdh	Rev	GCTGGGTCCCTCCTGTTTA
qPCR-bhik-2-42	Fw	CTTCTAAGTCATTAATTCCTGCAGCCCGGG
qPCR-bhik-2-42	Rev	TAACCCTCACTAAAGGGAACAAAAGCTGG
qPCR-bhik-2-37	Fw	ATCTTTTGATAATTCCTGCAGCCCGGG
qPCR-bhik-2-37	Rev	TAACCCTCACTAAAGGGAACAAAAGCTGG
qPCR-eGFP	Fw	GCGAGGGCGATGCCACCTACG
qPCR-eGFP	Rev	TCACCTCGGCGCGGGTCTTGT
qPCR-MER4-int exon-exon	Fw	CATGACGGTGGAAGGCAGATAAC
qPCR- MER4-int exon-exon	Rev	GCTACTTACCTAGGAATGGGTCTC
qPCR- MER4-int exon-intron	Fw	CATGACGGTGGAAGGCAGATAAC
qPCR- MER4-int exon-intron	Rev	ATGTGCCCAATGCTAGGAATG
qPCR-HERVK3-int exon-exon	Fw	GACAGCCACAAGTGGCAC
qPCR- HERVK3-int exon-exon	Rev	CGGTTTATCCATCTCCTTATATCC
qPCR- HERVK3-int exon-intron	Fw	GACAGCCACAAGTGGCAC
qPCR- HERVK3-int exon-intron	Rev	TTAGGACACTGACCTTATATCCTC

Table S5. Statistics for PAR-CLIP from Fig. 4L-M.

	Median Log2FC			
	LTRs	LINEs	Introns	Exons
Z18^{fl} versus Clover	1.38	1.86	1.78	0.73
Z18^{trunc} versus Clover	0.96	1.48	1.22	0.53

	P values compared to exons (Wilcoxon)		
	LTR	LINE	Introns
Z18^{fl} versus Clover	9.92E-179	0.00E+00	0.00E+00
Z18^{trunc} versus Clover	3.03E-100	0.00E+00	0.00E+00

Table S6. Z18 motifs from PAR-CLIP

Motif ID	Motif
HXA13_HUMAN.H11MO.0.C	GDTTTTATTGG
HXB13_HUMAN.H11MO.0.A	TTTTATDGSH
TBP_HUMAN.H11MO.0.A	MTATAAAARS
FOXA1_HUMAN.H11MO.0.A	TGTTTACWYWGB
STAT2_HUMAN.H11MO.0.A	RRGRAAAHGAAACTGAAAV
HXA10_HUMAN.H11MO.0.C	KATRRTTTATGW
SOX5_HUMAN.H11MO.0.C	YATTGTTW
ALX1_HUMAN.H11MO.0.B	RTAATTRVATTA
IRF1_HUMAN.H11MO.0.A	RAAANWGAAASTGAAASYRR
PRRX2_HUMAN.H11MO.0.C	YTAATTR
FOXJ3_HUMAN.H11MO.0.A	TTGTTTATKKTTW
MEF2B_HUMAN.H11MO.0.A	DKKKCTATTTTTRG
